# Self-consistent numerical simulations for the formation and dynamics of solar prominences

**DOI:** 10.1038/s41550-026-02840-7

**Published:** 2026-04-22

**Authors:** Lisa-Marie Zessner, Robert H. Cameron, Sami K. Solanki, Damien Przybylski

**Affiliations:** Max-Planck-Institut für Sonnensystemforschung, Göttingen, Germany

**Keywords:** Solar physics, Computational astrophysics

## Abstract

Solar prominences are cool and dense plasma structures floating in the hot solar corona. They are ubiquitous features in the solar atmosphere, but their formation mechanism is still unclear. Here we perform comprehensive fully three-dimensional numerical simulations of prominence formation including the physics necessary to describe all atmospheric layers of the sun. With appropriate initial conditions for the magnetic field, solar prominences form self-consistently in the simulations. The formation starts by the random ejection of a dense plasma seed from the chromosphere into the corona. Subsequently, the prominence is built up by a combination of plasma injections from the chromosphere and condensation of inflowing coronal plasma. The prominence properties qualitatively match those of observed prominences. Our findings demonstrate the importance of the dynamics at and below the solar surface in the formation and evolution of solar prominences. This suggests that subsurface dynamics should also be considered in the study of prominence eruptions, which can be associated with coronal mass ejections.

## Abstract (web summary)

Self-consistent 3D simulations reveal how solar prominences can form and demonstrate dynamic structures that are consistent with observations. Plasma ejected from the surface creates cool structures in the hot solar atmosphere that continue to grow by coronal condensation.

## Main

Solar prominences are beautiful and complex objects in the solar atmosphere. As gas clouds that are two orders of magnitude cooler and denser than the surrounding hot solar corona, they appear in emission when observed at the solar limb and as dark filaments on the solar disk. Solar prominences are as diverse as they are common—they can have very different sizes, fine structures and underlying magnetic field configurations^1^. Even though the prominence plasma is very dynamic, prominences can form long-lived, stable structures, with lifetimes ranging from days to months. At the end of their lifetimes, they can disappear quietly or erupt. Erupting prominences are often associated with coronal mass ejections^2^, which can produce violent space weather effects when they reach Earth. When prominence material is present, the prominence mass can influence or trigger the eruption process^3^. Realistic models of the formation and evolution of solar prominences should thus also contribute to long-term space weather forecasts. In this Article, we present self-consistent simulations that include the relevant physical processes in all atmospheric layers guiding the formation and dynamics of solar prominences.

The solar atmosphere is filled with plasma with temperatures of about 5,800 K at the solar surface (the photosphere), 6,000–20,000 K in the overlying chromosphere and over a million degrees in the upper solar atmosphere (the corona)^4^. Turbulent convective motions continually generate and rearrange the magnetic field below the surface. These changes rearrange the magnetic field in the upper chromosphere and corona where it dominates the energetics. Such subphotospheric turbulent driving also strongly affects the dynamics and evolution of prominences but has so far only been included heuristically in prominence models^5^ and not in a self-consistent manner.

The cool and dense plasma in prominences is held up against gravity by magnetic fields^6,7^. For small prominences, it is possible to accumulate the prominence mass by in situ condensation from the coronal environment^8^, whereas massive prominences need an additional mass reservoir^9^. In this case, the plasma must be transported up from the lower-lying chromosphere. Several mechanisms have been proposed for this mass supply^10^. The most basic models are direct injection, levitation and condensation. In the direct injection model^11,12^, cool plasma is directly transported along the magnetic field lines from the chromosphere into the corona. In the levitation model^13–15^, emerging magnetic fields capture and lift the cool plasma on their way up to the corona. In the condensation model^9,16–18^, chromospheric gas is first heated and evaporated into the corona, where it then condenses again to form the cool prominence plasma. The evaporation–condensation model has been studied extensively in a number of numerical simulations of prominence formation^5,19–21^. Combinations of the three basic mechanisms have also been proposed^22–24^. Numerical simulations have also been able to reproduce some prominence properties such as fine structures, instabilities and oscillations^8,25–30^. Despite the large variety of existing prominence models, so far there is no simulation of a solar prominence that includes a self-consistent treatment of the upper part of the convection zone and the solar surface. The photospheric dynamics are expected to have an important influence on especially low-lying active-region prominences. We present the formation and properties of an active-region-like prominence in the three-dimensional (3D) radiative magnetohydrodynamic (MHD) code MURaM, which captures the most important physical processes to describe the photosphere, chromosphere and corona self-consistently. Our results show that the surface dynamics plays an important role in the formation and mass supply of the prominence.

## Numerical methods and simulation setup

Here we gain insights into the physics that drives the formation and the mass supply of solar prominences by performing numerical simulations. These simulations, carried out with the MURaM code^31–33^, capture the dominant physical processes in each layer of the solar atmosphere and are therefore self-consistent. MURaM is a box-in-a-star code that explicitly treats the energy transport in the different solar layers, including convection and small-scale dynamo action^34^ in the photosphere^31^, a non-equilibrium treatment of hydrogen and a multigroup scattering scheme in the chromosphere^32^, as well as optically thin radiative losses and field aligned heat conduction in the corona^33^. The MURaM code has been used to simulate sunspot umbrae, plage^31^, pores^35^, sunspots^36^, flux emergence and flares^37^.

**Extended Data Fig. 1:**
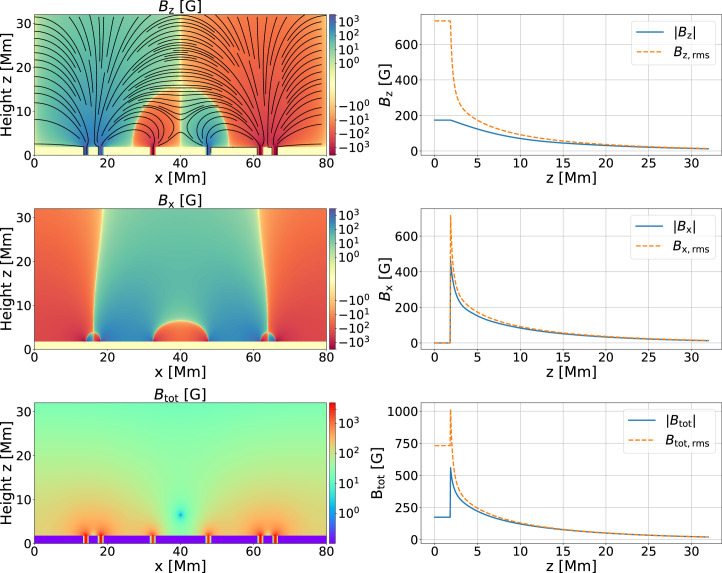
2D initial condition for the magnetic field configuration in Run I. The bottom boundary is here at *z* = 0, and the surface at *z* = 2 Mm. The polarity inversion line over which the prominence forms later in the 3D simulation is at *x* = 40 Mm. Left panels: 2D Maps of the vertical magnetic field component (top), the horizontal magnetic field component (middle) and the total magnetic field (bottom). The top left panel additionally shows magnetic field lines of the initial condition in black. Right panels: Horizontally averaged magnetic field strength in dependence of height over the bottom boundary for the vertical magnetic field component (top), the horizontal magnetic field component (middle) and the total magnetic field (bottom). The blue lines show the unsigned magnetic field and the dashed orange lines show the root mean square value of the magnetic field. The horizontal averages are taken over the whole box.

**Extended Data Fig. 2:**
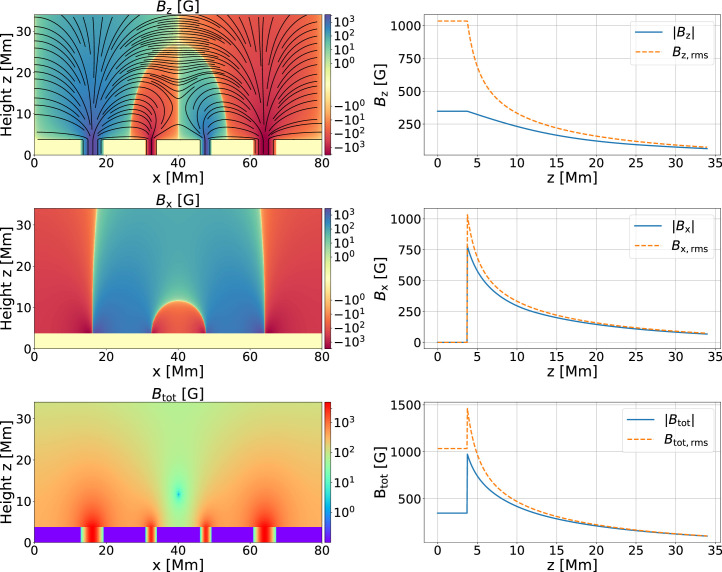
2D initial condition for the magnetic field configuration in Run II. The bottom boundary is here at *z* = 0, and the surface at *z* = 4 Mm. The polarity inversion line over which the prominence forms later in the 3D simulation is at *x* = 40 Mm. Left panels: 2D Maps of the vertical magnetic field component (top), the horizontal magnetic field component (middle) and the total magnetic field (bottom). The top left panel additionally shows magnetic field lines of the initial condition in black. Right panels: Horizontally averaged magnetic field strength in dependence of height over the bottom boundary for the vertical magnetic field component (top), the horizontal magnetic field component (middle) and the total magnetic field (bottom). The blue lines show the unsigned magnetic field and the dashed orange lines show the root mean square value of the magnetic field. The horizontal averages are taken over the whole box.

**Extended Data Fig. 3:**
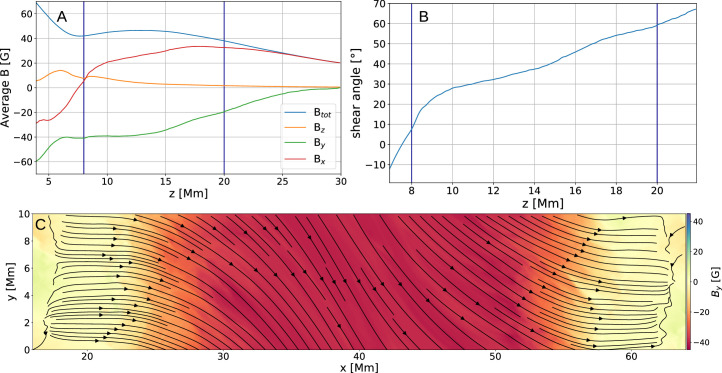
Magnetic field strength and orientation for the Shear run. **A**: Magnetic field components for the sheared setup of Run I, averaged over the polarity inversion line and a time frame of 10 minutes. The different curves show the total magnetic field (*B*_tot_, blue line), the vertical magnetic field component (*B*_z_, orange line) and the two horizontal magnetic field components (*B*_y_, green line, and *B*_x_, red line). The components *B*_*x*_ and *B*_*y*_ are used to determine the shear angle in panel **B**. **B**: Shear angle for the sheared setup of Run I in dependence of height above the surface, averaged over the polarity inversion line and a time frame of 10 minutes. The shear angle measures the angle between the *y* direction (along which the long axis of the prominence is located) and the horizontal direction of the magnetic field. The blue vertical lines indicate the lower and upper height limit between which the prominence mass is mainly found. The prominence can occasionally be higher than the indicated sizes. **C**: Example for the magnetic field component in the *y* direction for the sheared setup of Run I, taken along a horizontal slice. Magnetic field lines projected onto the plane are added in black. The height of the horizontal slice is 12 Mm above the surface.

The most commonly proposed magnetic field configurations for prominences are either a magnetic flux rope or a dipped arcade^6,7,38^. In either case, a region is present above the solar surface where the magnetic field lines are concave upwards, forming a dip. Since plasma is free to flow along field lines, such a dip is essential to support the prominence material against gravity. We chose a dipped arcade structure for the magnetic field configuration in our simulations. Starting with a potential field based on this (Extended Data Figs. 1 and 2), the resulting quadrupolar magnetic field structure is initially not sheared. We present three different simulations that are based on this initial condition, which we call Run I, Run II and Shear in the following. Run I and Run II are both based on the non-sheared, potential setup from the Extended Data Figs. 1 and 2. Run II has a higher magnetic field strength, a deeper convection zone and a slightly different bottom boundary condition compared with Run I (see the Methods for details on the implementation). To account for the high degree of shear that is normally observed for real prominences^1^, the third simulation is a sheared configuration based on the setup of Run I with an already built-up prominence (see the Methods and Extended Data Fig. 3 for a description). We chose the presented setups because the resulting prominences are governed by the same physical processes but have different appearances.

All runs were simulated with the local thermodynamic equilibrium (LTE) version of the MURaM code^33^. Run I and II were additionally run with the non-LTE (NLTE) version^32^, which includes a time-dependent treatment of hydrogen ionization and a scattering radiation transfer scheme in addition to the physics captured by the LTE version of the code (Methods). Results in the main text concentrate on the LTE results. The only exception to this is the presented approximated Hα emission, which uses the non-equilibrium hydrogen populations that are calculated in the NLTE simulations. Further NLTE results are shown in Supplementary Information section 1.

The lower boundary condition on the magnetic field pins the footpoints of the magnetic arcade. Boundary conditions are periodic in the horizontal directions and matched to a potential field at the upper boundary.

**Fig. 1:**
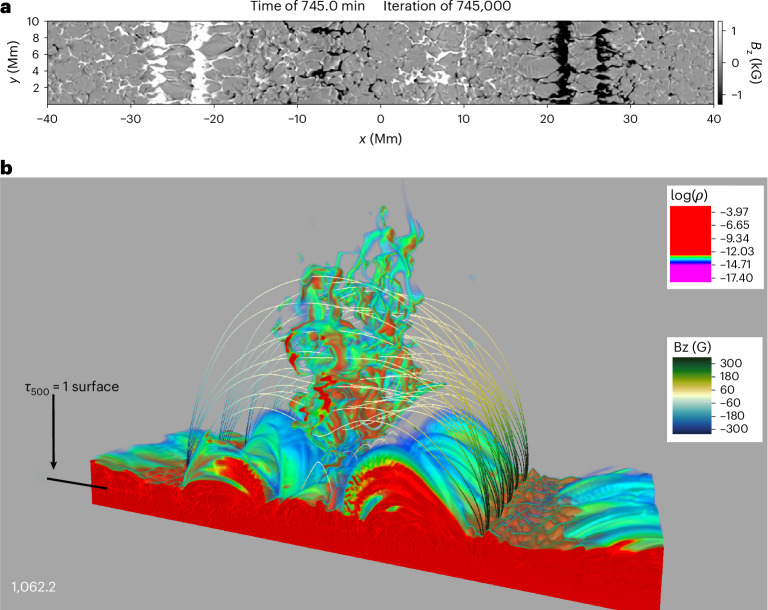
Visualization of the prominence and magnetic field structure for Run I. **a**, The vertical magnetic field at the *τ*_500_ = 1 surface at around 60 min after prominence formation starts. See Supplementary Video 1 for an an animation. **b**, A 3D rendering of the prominence density with a set of field lines to demonstrate the quadrupolar structure of the magnetic field. This snapshot shows the prominence 300 min after the formation starts. The colouring of the plasma (rainbow colourmap) shows the logarithmic gas density (in grams per centimetre cubed). For plasma with a density below 10^−14^ g cm^−^^3^, the opacity is set to zero for this image, such that the surrounding corona is not visible. The colouring of the magnetic field lines (blue–green colourmap) shows the value of the vertical magnetic field component. Bottom left: the number shows the time in minutes since the start of the simulation. The thick black line indicates the average height where *τ*_500_ = 1, roughly corresponding to the location of the photosphere. See Extended Data Fig. 5 for the corresponding images of Run II.

**Extended Data Fig. 4:**
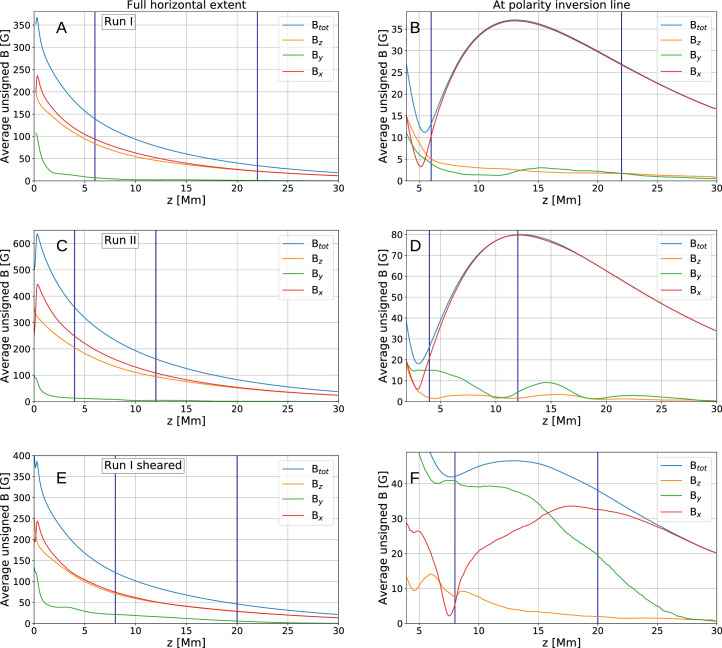
Magnetic field strength within the whole simulation box and at the location of the prominence. Magnetic field strength for Run I (**A**, **B**), Run II (**C**, **D**) and the sheared setup of Run I (**E**, **F**), averaged over a time frame of 50 minutes for Run I and Run II, and over 10 minutes for the sheared configuration. The chosen time frames start after the prominences are fully built up. The different curves show the total magnetic field (*B*_tot_, blue line), the vertical magnetic field component (*B*_z_, orange line) and the two horizontal magnetic field components (*B*_y_, green line, and *B*_x_, red line). The blue vertical lines indicate the lower and upper height limit between which the prominence mass is mainly found. The prominences can occasionally be higher than the indicated sizes. **A**, **C**, **E**: Horizontally averaged unsigned magnetic field in dependence of height over the surface. **B**, **D**, **F**: Unsigned magnetic field averaged along the polarity inversion line as a measurement for the magnetic field in the prominence core, in dependence of height over the surface. The height for the sheared setup is higher than for Run I because the shearing process lifts the height of the magnetic dips.

**Extended Data Fig. 5:**
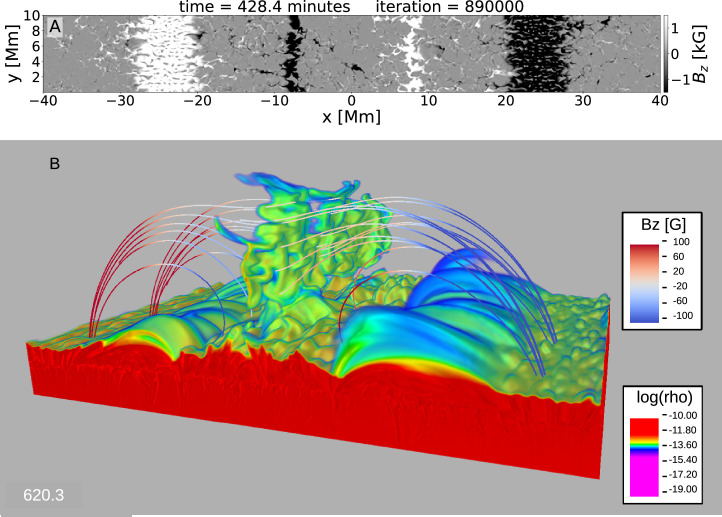
Visualization of the prominence and magnetic field structure for Run II. Equivalent to Fig. 1 of the main text, showing the configuration of the magnetic field for Run II. **A**: Magnetic field at the *τ*_500_ = 1 surface at around 30 min after prominence formation starts. Supplementary Video 9 shows an animation of this panel. **B**: 3D rendering of the prominence density (rainbow coloured) with a set of field lines to demonstrate the quadrupolar structure of the magnetic field (blue-red field lines). This snapshot shows the prominence 200 min after the formation starts. The colouring of the plasma (rainbow colourmap) shows the logarithmic gas density (in g/cm^3^). For plasma with a density below 10^−14^ g/cm^3^, the opacity is set to zero for this image, such that the surrounding corona is not visible. The colouring of the magnetic field lines (blue-red colormap) shows the value of the vertical magnetic field component. The number at the bottom left shows the time in minutes from the start of the simulation.

Except at the lower boundary, the magnetic field evolves in accordance with the induction equation (equation (4)). Figure 1a shows the photospheric magnetic field for Run I after the initial magnetic field and interior convection have been evolved to a statistically stationary state. The imposed quadrupolar structure is still clear (positive polarity on the left hand side, a ridge of negative polarity at about −8 Mm, a ridge of positive polarity at +8 Mm and negative polarity field to the right). The effect of the turbulent convection is seen in the fine structure. Supplementary Video 1 shows that the magnetic structure is constantly evolving. Figure 1b shows a 3D rendering of the prominence density and magnetic field lines for Run I. We can see how the quadrupolar structure from the magnetogram translates to a dipped magnetic arcade in the corona. The simulated prominence is hanging in the magnetic dips of this arcade, above the polarity inversion line. Due to the magnetic null point in our setup, the magnetic field strength in the dips first increases, then decreases with increasing height above the surface (Extended Data Fig. 4). Extended Data Fig. 5 equivalently shows the magnetic field configuration for Run II.

The magnetic field strength within the prominence is in the range of ~15–37 G for Run I, ~30–80 G for Run II and ~38–45 G for the Shear simulation, as shown in Extended Data Fig. 4. The average field at these heights is ~50–120 G in Run I and the sheared setup, and ~160–360 G in Run II. This is much higher than the field in the prominence itself. The magnetic field values within the prominences are not as high as the very strongest measured values for active-region prominences^39^ but notably higher than the values for most quiescent prominences^1^. Furthermore, the simulated prominences are low-lying (4–7 Mm over the surface) and have heights of 6–20 Mm that are similar to prominences in active regions. Owing to these dimensions and the surrounding magnetic field strength, we classify the simulated prominences as active-region prominences. However, magnetic field values of 30 G and higher are also reasonable for intermediate prominences^40^ and have even been observed in some quiescent prominences^41^. We therefore compare the fine structure of the simulated prominences with all prominence types.

In the sheared setup, the shear angle of the magnetic field with respect to the long prominence axis is in the range of 15–60° within the prominence (Extended Data Fig. 3), which makes the shear angle in the central part of the prominence body comparable to observed values of around 35° (ref. ^1^).

## Prominence formation and mass supply

**Fig. 2:**
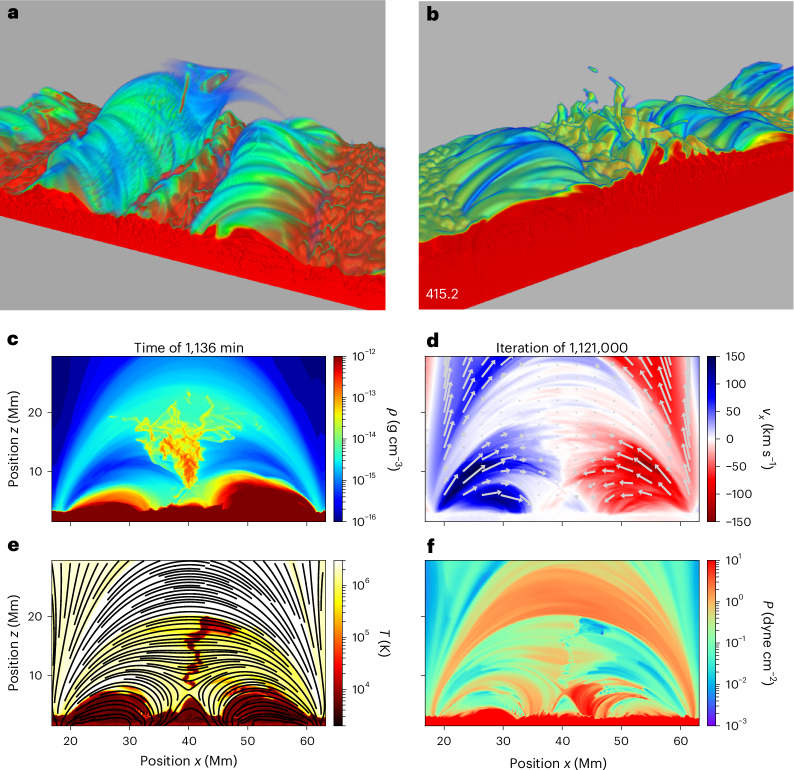
Formation of the simulated prominences in two steps. **a**, A 3D rendering of the prominence density, showing a snapshot during the formation phase in Run I. A seed of cool plasma is transported along the loops on the left side into the magnetic dips. **b**, A 3D rendering of the prominence density, showing a snapshot during the formation phase in Run II. The initial cool seed is here ejected into the dips from the polarity inversion line. Bottom left: the number shows the time in minutes since the start of the simulation. **c**–**f**, A vertical slice through one snapshot when the prominence in Run I is fully built up. It shows how the prominence is fed via condensation of hot plasma that is flowing along the magnetic field lines onto the cool prominence structure, driven by a pressure drop at the cool prominence material. The density (**c**) and horizontal velocity (**d**) are averaged over the current line-of-sight. For better visibility, the temperature (**e**) and pressure (**f**) are taken along one vertical slice of the box. The arrows in **d** show the line-of-sight averaged velocity field. Line-of-sight averaged magnetic field lines are added to **e**. Supplementary Videos 2, 3 and 4 show an animation for **a**, **b** and **c**–**f**, respectively. Supplementary Video 5 shows the equivalent of **c**–**f** for Run II.

Prominences form spontaneously in this simulation. The formation is initiated by random chromospheric motions (driven by the turbulent convection) that inject a small blob of cool chromospheric material into the magnetic dips. For Run I, this happens at 670 min and for Run II at 410 min after the start of the 3D simulation. The ejection of this seed of dense material into the corona is caught on the Supplementary Videos 2 and 3. In Run I, this blob is supplied by a cool flow along the small magnetic loops on the left side (Fig. 2a and Supplementary Video 2). In Run II, this blob is ejected from below the magnetic dips (Fig. 2b and Supplementary Video 3). Once in the corona, two processes cause the blob to grow in mass.

One of these mass supply mechanisms is caused by a radiative instability that occurs as the cool prominence material (and its surrounding transition region) loses energy by radiation (Supplementary Figs. 1 and 2). This causes the pressure to drop and drives a syphon flow^9^, where plasma flows onto the prominence structure along the magnetic field lines passing through the corona (Fig. 2c–f and Supplementary Fig. 3). The resulting syphon flow can be seen in Fig. 2d as an inflow of plasma in the horizontal velocities as soon as the initial plasma blob settles into the magnetic dip (Supplementary Videos 4 and 5). Hot plasma (Fig. 2e) flows up along the field lines on both sides and condenses onto the prominence structure. This mass supply of the simulated prominence therefore corresponds to the condensation model^8,19^. Supplementary Figs. 4 and 5 show that thermal instabilities play a role in the transition region around the prominence and the cool injected seeds. This aligns with what has been found in previous work^18^. In contrast to models where the prominence forms solely by condensation, the condensation process in this simulation does not start the prominence formation process, as it sets in only after the first dense seed has been injected from the chromosphere into the corona.

The second process that supplies mass is the continued ejection of dense plasma blobs from the chromosphere, which happens from time to time throughout the whole simulation. This mechanism is the same as that which is responsible for supplying the initial prominence seed. The ejections mainly happen from below the prominence for Run II and from the magnetic side loops next to the prominence for Run I (Supplementary Videos 2 and 3). Supplementary Section 3 takes a closer look on the dynamics of an injection event in Run II. For the chosen event as shown in Supplementary Fig. 6 (Supplementary Video 10), the chromospheric plasma is injected into the corona from below the null point. Around the time of the injection, flux cancellation occurs around the footpoints of the loops that lie under the null point (Supplementary Fig. 9 and Supplementary Video 14). Above the surface, reconfigurations of the magnetic field are visible while the chromospheric plasma is accelerated upwards from the chromosphere. Both the Lorentz force and the pressure gradient force contribute to the dynamics of the accelerated plasma (Supplementary Fig. 7 and Supplementary Video 11). Through rearrangements of the magnetic field at the null point, the chromospheric plasma reaches the corona and contributes to the prominence mass (Supplementary Fig. 8 and Supplementary Videos 12 and 13). Once in the corona, the material is supported against gravity by the Lorentz force (Supplementary Fig. 10). Further examples of ejected blobs can be seen in Supplementary Videos 6 and 7, in the left and top right panels. This mass supply mechanism is ubiquitous in the simulation. In Run II, for example, at *t* ≈ 560 min, *y* ≈ 6–8 Mm and *x* ≈ 40 Mm, we see a large ejection from the bottom, which supplies part of its mass to the prominence, and a small ejection supplying mass at *t* ≈ 624 min, *y* ≈ 4–6 Mm and *x* ≈ 40 Mm. However, not all of these events supply mass to the prominence: a third example event occurs at *t* ≈ 523 min, *y* ≈ 1 Mm and *x* ≈ 40 Mm that only disturbs the prominence before falling back to the chromosphere. Supplementary Figs. 11 and 12 demonstrate that only sufficiently slow blobs are successful in building up the prominence.

The syphon flow (condensation) and turbulent injections both contribute mass to the prominence throughout the simulation. For both simulations, the turbulent injection has a higher contribution to the mass supply (~58–82 %) than the condensation mechanism (~18–42 %) (see Supplementary Figs. 13 and 14 for more details).

## Prominence properties and dynamics

**Fig. 3:**
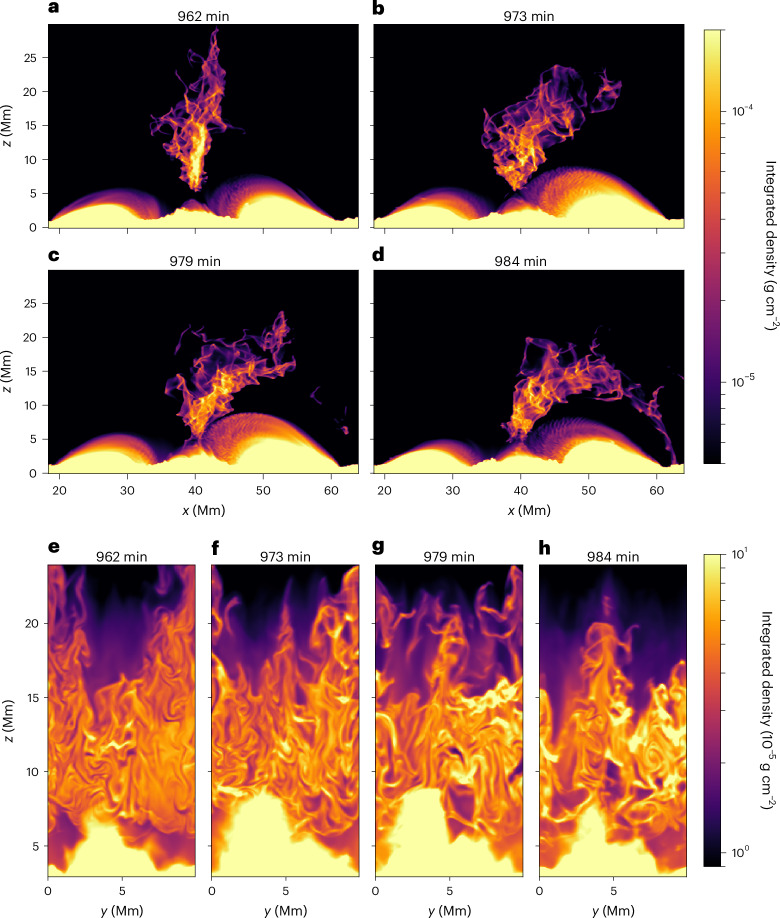
Prominence dynamics seen from two directions. **a**–**d**, Integrated density through the prominence from the front (integration along the *y* axis) for a series of four snapshots: a straight state of the prominence (**a**), followed by a draining event to the right in the next three snapshots (**b**–**d**). **b**–**d**, The prominence in three separate time steps during the draining event. **e**–**h**, Integrated density through the prominence from the side (integration along the *x* axis) for the same time series as in **a**–**d**. Supplementary Videos 6–8 show an animation of this figure for Run I, Run II and the Shear run.

The mass supplied to the prominence by injection and condensation is partly drained away again. Supplementary Videos 6–8 show that the prominence structure is continuously swaying from side to side along the *x* axis. During this motion, from time to time, cool plasma rains down along the magnetic field lines from the top of the prominence to the solar surface. This is possible because the prominence gas in our dipped arcade setup is not confined at the sides, such that the mass can in principle drain down at every point along the long prominence axis. Observations often show prominence mass draining in the context of eruptions^42^, but draining events during oscillatory motions have also been observed, in general agreement to what we find^43^. The prominence is slightly tilted during the draining process. An example of a draining event is shown in Fig. 3. As soon as the draining stops, the prominence straightens and the syphon flow sets in again. Alternating between mass supply by condensation and injection and mass loss by draining, the prominence is dynamically stable over a timescale of hours. For Run I, the prominence structure is stable over the whole simulation time, which is 12 h from the formation time. The prominence mass increases for the first 200 min after the start of the formation, then stays approximately constant for the next 200 min and starts to increase slightly towards the end of the simulated time frame (Supplementary Fig. 14). For Run II, the prominence structure is not stable. After the formation, the prominence grows in mass for the next 230 min. After this time, the prominence material drains down completely in a big draining event. The formation of a new prominence starts again 134 min after complete disappearance. This new prominence disappears and reforms again before we stop the simulation (Supplementary Fig. 14). The formation and mass supply procedure is the same for all three formations. As observed in real prominences^44^, the total mass that circulates through the prominence is several times the prominence mass for both runs, which shows how dynamic the structure is (Supplementary Section 4).

In the prominence cores, we get temperatures of 6,000–8,000 K and densities of 10^−13^ to 10^−12^ g cm^−3^, which are comparable to observations^1,45^. Also the pressure values (Supplementary Fig. 3) are in line with the values cited in ref. ^1^.

**Fig. 4:**
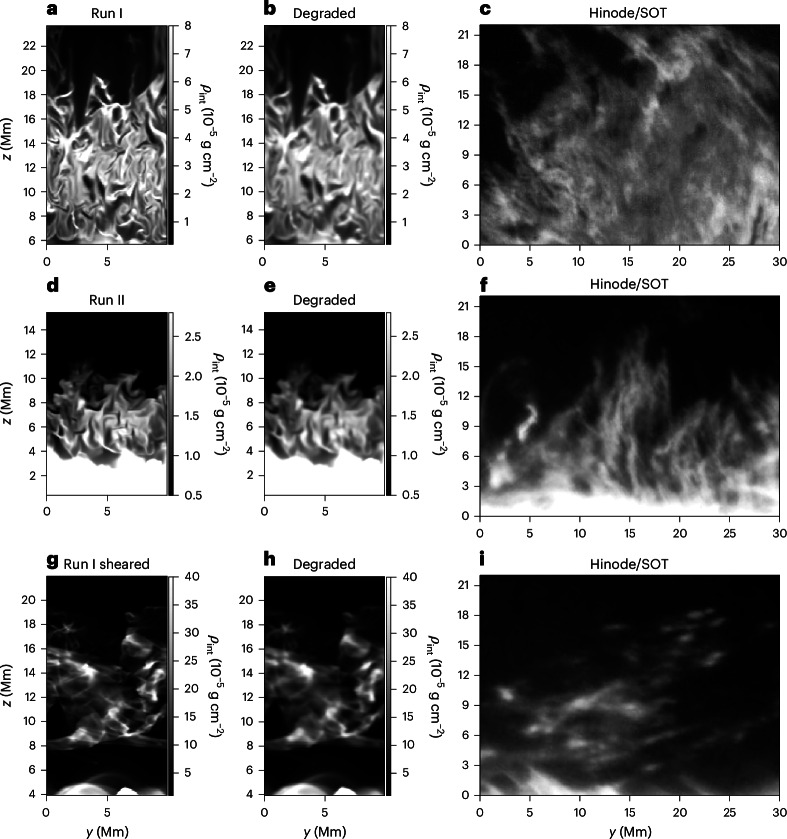
Fine structure of the three simulated prominences in comparison with Hinode/SOT observations. **a**,**d**,**g**, Integrated density in the simulation from the side for Run I (**a**), Run II (**d**) and the Shear simulation (**g**). **b**,**e**,**h**, Graphs of **a** (**b**), **d** (**e**) and **g** (**h**) degraded to Hinode/SOT resolution. **c**,**f**,**i**, Hinode/SOT observations of individual prominences above the limb. The observations were taken on 26 July 2013 (**c**), 25 June 2010 (**f**) and 16 October 2014 (**i**). The shown length scales along the *y* and *z* axis were obtained by using a constant factor of 725 km arcsec^−1^ for all observations. The observed images were rotated such that the local plane of the solar surface is approximately parallel to the *y* axis of the simulations.

Figure 4 shows the general prominence appearance in integrated density for the three simulation runs in comparison with three observations from the Hinode Solar Optical Telescope (SOT)^46^ that resemble the structure of the simulated prominences. For a better comparison, the simulated images were degraded using a Gaussian filter with a FWHM of 0.3 arcsec. The fine-structure of Run I (Fig. 4a,b) is more turbulent and resembles the diffuse structure of the observed prominence in Fig. 4c. The left part of the observation especially shows a similar mixture of vertical and horizontal structures, which can often be observed in intermediate prominences^40^. In addition to this turbulent pattern, Run II (Fig. 4d,e) shows a few elongated, more vertical structures that resemble the observation in Fig. 4f. These vertical structures are generally more typical for quiescent prominences^40^. For both Run I and Run II, the magnetic arcade is not sheared, which is the reason they do not feature the pronounced horizontal pattern that is typical for active-region prominences on the limb. Run I has an overall lower magnetic field, which allows the plasma to spread more freely across the magnetic field lines (in the *y* direction) and produces a more turbulent looking structure compared with Run II. The sheared setup of Run I (Fig. 4g,h) starts to show more horizontal structures compared with the original fine structure of Run I, which is more in line with what is expected from an active-region prominence^47^. Along the polarity inversion line, the magnetic field configuration in this run is strongly sheared, which makes the magnetic field orientation mostly horizontal in the *y*–*z* plane, leading to horizontal structures. The shear strength decreases with height and is in the range of 15° to 60° (Extended Data Fig. 3). As the magnetic field value within our prominences is still on the lower side for an active-region prominence, we consider it reasonable to compare the simulated fine structures with all prominence types.

**Fig. 5:**
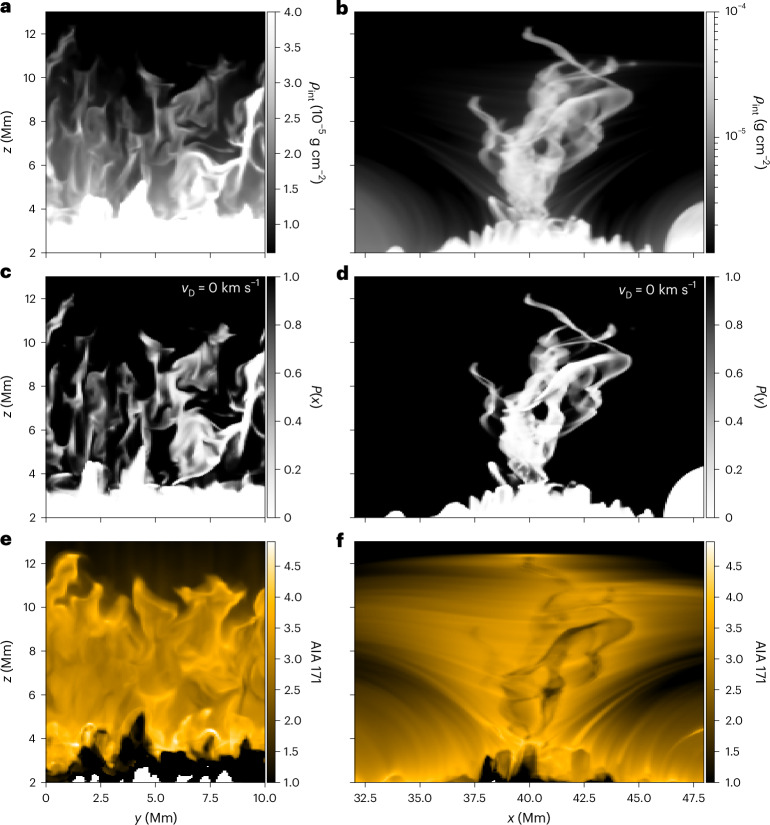
Appearance of the prominence in Run II NLTE in Hα and AIA 171. **a**–**f**, A comparison of integrated density (**a** and **b**), Hα emission in the line core (**c** and **d**) and AIA 171 emission (**e** and **f**) for the prominence view in Run II. **a**, **c** and **e** show the prominence from the side (line-of-sight along the *x* axis), and **b**, **d** and **f** show the prominence from the front (line-of-sight along the *y* axis). Following ref. ^48^, the Hα emission is approximated by calculating an integrated hydrogen opacity that uses the non-equilibrium hydrogen populations from the NLTE version of the MURaM code. The shown emission is taken in the Hα line core (that is, at a Doppler velocity of *v*_D_ = 0). The AIA 171 emission is calculated following ref. ^49^ and takes absorption into account. Extended Data Fig. 6 equivalently shows the appearance of the prominence in Run I NLTE.

**Extended Data Fig. 6:**
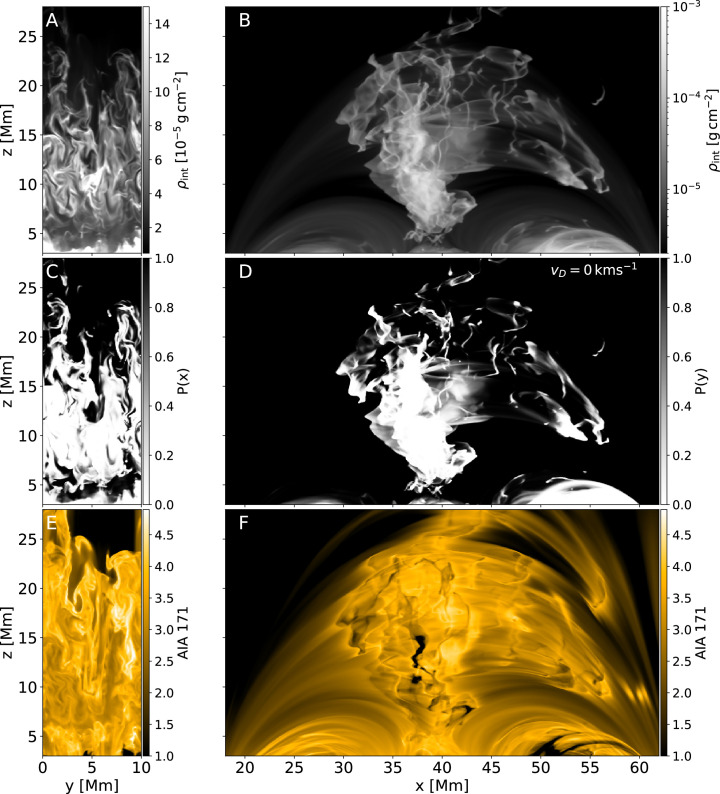
Appearance of the prominence in Run I NLTE in the H*α* line core and in AIA 171. The figure shows the comparison of integrated density (**A**, **B**), H*α* emission in the line core (**C**, **D**) and AIA 171 emission (**E**, **F**) for a snapshot from Run I NLTE (see also Fig. 5). Due to the saturation of the H*α* proxy in the line core, some of the substructures visible in the integrated density (panels **A** and **B**) are washed out in the H*α* images (panels **C** and **D**). For Run I, the saturation in H*α* is clearer, especially for the bottom and middle part of the prominence, because the prominence is thicker and more massive compared to the prominence in Run II. The less dense structures in the upper part of the prominence look more pronounced compared to the structures in integrated density. The prominence fine structure that is visible in integrated density and in H*α* can be partly seen in absorption in AIA 171. For comparison to the appearance in the H*α* line core, we show an image of Run I in the blue wing of H*α* at a Doppler velocity of *v*_*D*_ = 12 km s^−1^ in Extended Data Fig. 7.

**Extended Data Fig. 7:**
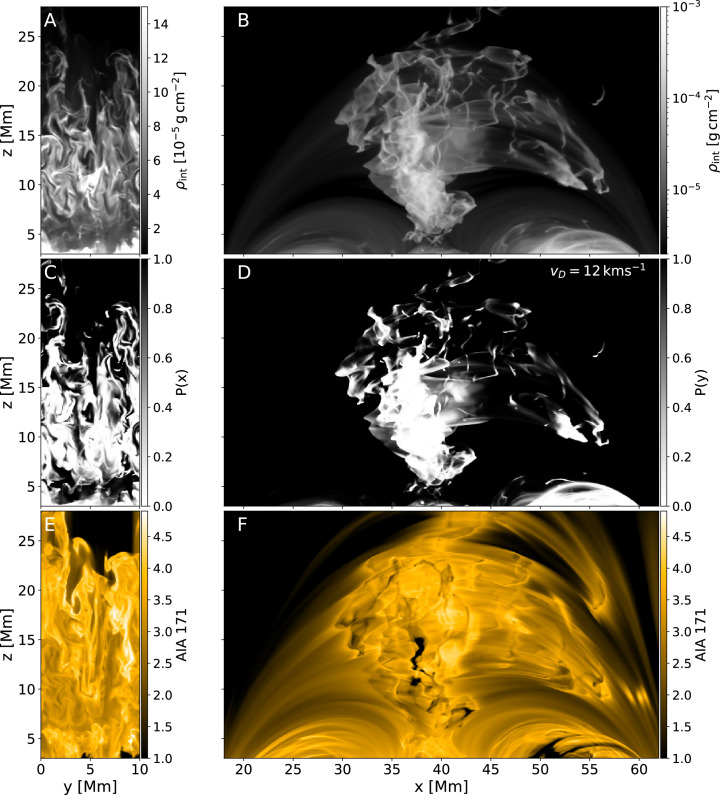
Appearance of the prominence in Run I NLTE in the blue wing of H*α* and in AIA 171. The figure shows the comparison of integrated density (**A**, **B**), H*α* emission in the blue wing (**C**, **D**) and AIA 171 emission (**E**, **F**) for a snapshot from Run I NLTE. The figure is the same as Extended Data Fig. 6, but the H*α* emission (panels **C** and **D**) is here shown for the blue wing at a Doppler velocity of *v*_*D*_ = 12 km s^−1^. In the side view (panel **C**), the fine structure is less saturated and thus more visible compared to the structure seen in the H*α* line core (panel **C** in Extended Data Fig. 6).

In addition to the appearance of the prominence in integrated density as shown in Fig. 4. Figure 5 and Extended Data Figs. 6 and 7 show the appearance of the prominences for Run I and Run II in Hα and AIA 171 emission for the prominence view. We calculated the Hα images following the approximative approach developed in ref. ^48^. To calculate the synthesized Hα emission, these authors used an integrated hydrogen opacity that takes the non-equilibrium populations from the MURaM NLTE simulations into account. We follow this method and use the NLTE simulations of Run I and Run II with the corresponding non-equilibrium hydrogen populations. Figure 5 and Extended Data Fig. 6 show the resulting appearance for the prominence view in the line core of the Hα proxy (at a Doppler velocity of *v*_D_ = 0 km s^−1^) in comparison with the appearance in integrated density. The structures visible in Hα (Fig. 5c,d) are similar to the structures seen in integrated density (Fig. 5a,b). Owing to the saturation of the Hα proxy in the line core, some of the substructures visible in the integrated density are washed out in the Hα images. In the side view (Fig. 5c), the fine structure looks more pronounced in Hα compared with the smoother appearance in integrated density (Fig. 5a). For comparison to the appearance in the H*α* line core, we show an image of Run I in the blue wing of Hα at a Doppler velocity of *v*_D_ = 12 km s^−1^ in Extended Data Fig. 7. The AIA 171 emission is calculated as described in ref. ^49^. The method used here also includes absorption. From the front (Fig. 5f), the prominence structure that is visible in integrated density and Hα can be seen in absorption in AIA 171. From the side (Fig. 5e), the prominence structure looks relatively smooth, with some visible fine structure that appears slightly darker. No cavity structure is visible in AIA 171, in contrast to what is often seen in observations^50^. We plan to study if this changes in the future when moving to other magnetic field configurations.

**Fig. 6:**
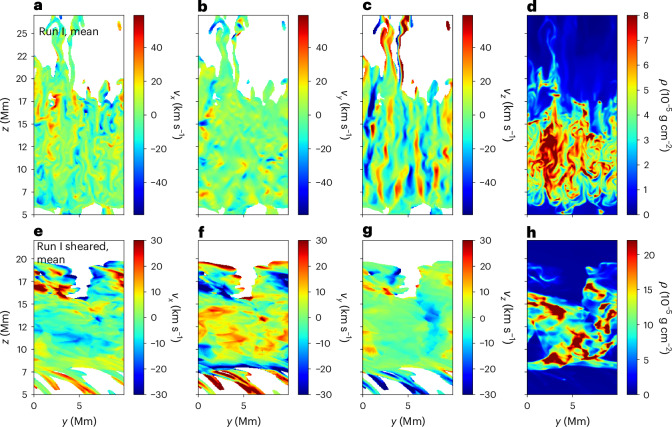
Flow structures for one snapshot of the non-sheared and the sheared setup of Run I. **a**–**d**, Velocities in the *x* (**a**), *y* (**b**) and *z* direction (**c**) and integrated plasma density (**d**) along a vertical slice in the *y*–*z* plane for Run I. For all quantities apart from the density, only prominence plasma with *ρ* > 10^−14^ g cm^−3^ is shown. All velocities are averaged along the line-of-sight (here, the *x* axis). **e**–**h**, Velocities in the *x* (**e**), *y* (**f**) and *z* direction (**g**) and integrated plasma density (**h**) along a vertical slice in the *y*–*z* plane -for the sheared setup of Run I. All velocities are averaged along the line-of-sight (here, the *x* axis). The density in **d** and **h** is integrated along the *x* axis.

Figure 6 shows flow patterns of the prominence fine structure for one snapshot of Run I and the Shear simulation. The velocities are averaged over the line-of-sight. For both simulations, counterstreaming flows are visible. In Run I (Fig. 6a–d), we see vertically oriented up-and-downflows side by side (Fig. 6c). Moreover, along the *x* direction (Fig. 6a), flows in both directions are visible. Similar to the fine structure in Fig. 4, these counterstreaming flows develop a strong component along the *y* axis in the Shear setup (Fig. 6e–h) owing to the build-up of a *y* component in the magnetic field during the shearing process. In observations, a variety of flows is seen, often including these counterstreaming flows as a striking feature^51^. Quiescent prominences mostly show vertical^40^ and active-region prominences horizontal flows^47^.

## Summary

We have simulated the formation and properties of a solar prominence in MURaM for three different configurations of our setup. We find that prominence formation happens self-consistently by the random injection of a dense seed into the magnetic dips. The subsequent mass build-up of the prominence happens via a mixture of the injection and the condensation mechanism. In contrast to other simulations that show the formation of prominences via condensation^19–21^, condensation does not start the formation process in our simulation but only sets in after the first dense seed settles in the magnetic dips via injection (see Supplementary Section 5 for a discussion). This shows the importance of a self-consistent connection of the solar corona with the underlying photosphere and chromosphere for the formation of low-lying solar prominences. Even for larger prominences, the injection process may initiate prominence formation if the underlying magnetic field configuration rises from low altitudes into the corona during the prominence formation process. In this case, the presented model of injection followed by condensation would not be limited to low-lying prominences. Instead, it could also feed larger prominences by providing the initial dense material via injection before the magnetic structure rises higher into the atmosphere. After the initial seed has been provided, the prominence can grow via condensation at larger heights.

The formation and dynamics of the simulated prominences are similar for all runs, whereas the sizes and appearances in the fine structure differ. This shows that different-looking prominences can form in our simulations despite the simple underlying magnetic field configuration. We see similarities to observations of different prominence types in the fine structure, whereas our sheared setup is more in line with the fine structures observed in active-region prominences than the non-sheared setups. In the future, we plan to set up more realistic magnetic field configurations, such as a magnetic flux rope, to study the differences in the structure and dynamics compared to our current setups and to do more detailed comparisons with observations.

## Methods

### The MURaM code

MURaM is a 3D box-in-a-star radiative MHD code incorporating the physics to simulate the solar photosphere^31^, chromosphere^32^ and corona^33^. MURaM solves the non-ideal MHD equations in 3D Cartesian coordinates

1 $$\frac{{{\partial }}\rho }{{{\partial }}t}=-\nabla \cdot (\rho {\bf{v}})$$

2 $$\frac{{{\partial }}\rho {\bf{v}}}{{{\partial }}t}=-\nabla \cdot \left(\rho {\bf{v}}{\bf{v}}\right)-\nabla p+\rho {\bf{g}}+{{\bf{F}}}_{{\rm{L}}}+{{\bf{F}}}_{\mathrm{SR}}$$

3 $$\frac{{{\partial }}{E}_{\mathrm{HD}}}{{{\partial }}t}=-\nabla \cdot \left[{\bf{v}}\left({E}_{\mathrm{HD}}+p\right)+q\frac{{\bf{B}}}{ B }\right]+\rho {\bf{v}}\cdot {\bf{g}}+{\bf{v}}\cdot {{\bf{F}}}_{{\rm{L}}}+{\bf{v}}\cdot {{\bf{F}}}_{\mathrm{SR}}+{Q}_{\mathrm{rad}}+{Q}_{\mathrm{res}}$$

4 $$\frac{{{\partial }}{\bf{B}}}{{{\partial }}t}=\nabla \times ({\bf{v}}\times {\bf{B}}),$$

where *ρ* is density, **v** is velocity, *p* is pressure, **g** is the gravitational acceleration, **B** is the magnetic field, **F**_L_ is the numerically computed Lorentz force (see equation (7)), ${E}_{\mathrm{HD}}={E}_{\mathrm{int}}+\frac{1}{2}\rho {v}^{2}$ is the hydrodynamic energy, *Q*_rad_ is the radiative cooling/heating, *Q*_res_ is the resistive heating from the diffusion scheme, *q* is the Spitzer heat flux and **F**_SR_ is the semi-relativistic (Boris) correction, used to prevent severe time-step constraints (see refs. ^32,33^ for details). The term *q***B**/*B* describes the conductive heat flux along the magnetic field lines, whereas the Spitzer heat flux *q* is calculated via

5 $$\frac{{{\partial }}q}{{{\partial }}t}=\frac{1}{\tau }\left(-{f}_{\mathrm{sat}}\sigma {T}^{\frac{5}{2}}\left(\frac{{\bf{B}}}{ {B} }\cdot \nabla \right)T-q\right).$$

The prefactor

6 $${f}_{\mathrm{sat}}={\left(1+\frac{| \sigma {T}^{\frac{5}{2}}(\widehat{{\bf{b}}}\cdot \nabla )T| }{1.5\rho {c}_{{\rm{s}}}^{3}}\right)}^{-1}$$

takes the saturation of the conductive heat flux into account^52,53^ (see ref. ^33^ for details). *σ* = 10^−6^ erg cm^−1^ s^−1^ K^−7/2^ is the Spitzer heat conductivity, and *c*_s_ is the sound speed.

Equations (1)–(4) describe the conservation of mass (1), momentum (2) and energy (3), as well as the evolution of the magnetic field, following the induction equation (4). A hyperbolic divergence cleaner is applied to fulfil ∇⋅**B** = 0 (ref. ^54^). There is no explicit treatment for viscosity and magnetic resistivity. Only numerical diffusivities are used as described in refs. ^32,33^ and ref. ^55^. Numerical diffusive fluxes are calculated from a slope-limited diffusion scheme^55^. Fourth-order hyperdiffusion terms are added in the vertical direction to $\log \rho$, internal energy density *ϵ* = *E*_int_/*ρ*, as well as the vertical components of the magnetic field and the velocity field (*B*_*z*_,*v*_*z*_). In the energy conservation equation (3), the hydrodynamic energy ${E}_{{\rm{HD}}}={E}_{{\rm{int}}}+\frac{1}{2}\rho {v}^{2}$, the sum of internal and kinetic energy, is used instead of the total energy to prevent numerical errors in regions where the magnetic energy is much larger than the hydrodynamic energy. Heating resulting from the diffusion scheme is included as ${Q}_{{\rm{res}}}$. The viscous heating is not directly included in the energy conservation because energy that is removed from the kinetic energy by viscous heating is added to the internal energy.

The Lorentz force is numerically computed by

7 $${{\bf{F}}}_{{\rm{L}}}=\frac{{f}_{{\rm{A}}}}{4{\rm{\pi }}}\nabla \cdot \left({\bf{B}}{\bf{B}}-\frac{1}{2}I{\bf{B}^2}\right)+\frac{1-{f}_{{\rm{A}}}}{4{\rm{\pi }}}\left(\nabla \times {\bf{B}}\right)\times {\bf{B}},$$

with identity matrix *I*. The factor ${f}_{{\rm{A}}}=\frac{1}{\sqrt{1+{\left({v}_{{\rm{a}}}/{c}_{\max }\right)}^{4}}}$ describes the limitation of the Alfvén velocity *v*_a_ that is used to prevent time-step constraints. For the reduction of the Alfvén velocity a semi-relativistic treatment with reduced speed of light is used (Boris correction). It introduces a force term **F**_SR_ in the momentum and energy equation (see ref. ^33^).

There are currently two different versions of the MURaM code: the coronal extension^33^ (also called MURaM-CE) and the chromospheric extension^32^ (also called MURaM-ChE). The coronal extension includes optically thin losses and thermal conduction along the field lines. It treats the radiation in LTE. The chromospheric extension^32^ covers all that the coronal extension includes, and additionally includes non-LTE (NLTE) effects in the chromosphere. In comparison with the coronal extension, the chromospheric extension additionally includes a time-dependent treatment of hydrogen ionization, an improved treatment of radiative losses in the chromosphere and a scattering multigroup radiation transfer scheme. Here, we distinguish between LTE and NLTE runs. We call it LTE when using the coronal extension only, whereas the NLTE runs also use the chromospheric extension.

Pressure, temperature and electron number are calculated from density and internal energy *E*_int_ using a pre-tabulated equation of state (EoS). In the LTE runs, an LTE EoS is used for the plasma in all regimes. The LTE EoS is tabulated using the FreeEoS code^56^ and the abundances of ref. ^57^. The NLTE runs additionally include an EoS with a non-equilibirum treatment for hydrogen and *H*_2_ molecules. This non-equilibrium EoS is used for the near photosphere and atmosphere, where *p* < 2 × 10^5^ dyne cm^−2^. The two methods are smoothly joint at this pressure^32^. All non-hydrogen atoms are treated in LTE, also for low pressures. The system of hydrogen rate equations includes the ground state, four excited states and the continuum.

MURaM includes different radiative transfer treatments for the different physical regimes of the solar atmosphere. For the photosphere, where the populations of atomic levels are dominated by collisions, the time-independent LTE radiative transfer equation is solved. A multigroup method is used to calculate the photospheric radiation field^58,59^. In the chromospheric extension^32^ a scattering term is added in the source function, following the prescription of ref. ^60^ and ref. ^61^. For the optically thin gas in the corona, optically thin losses are calculated from tabulated losses by an overlap interval approach^33^. The transition between the two approaches is defined by thresholds in optical depth and pressure (see ref. ^32^ for further details). Two different recipes are implemented for the optically thin losses: a Chianti loss function^62^ and a Carlsson and Leenaarts description^63^. The latter includes NLTE models for H I, Ca II and Mg II line losses as well as optically thin losses. The chromospheric extension furthermore includes extreme ultraviolet (EUV) back-heating of the chromospheric plasma, following ref. ^63^. The resulting radiative heating/cooling term in equation (3) is therefore *Q*_rad_ = *Q*_RT_ + *Q*_thin_ + *Q*_H_ + *Q*_Mg_ + *Q*_Ca_ + *Q*_back_ in the NLTE simulations, with optically thin losses *Q*_thin_, back-heating *Q*_back_, line losses *Q*_H_, *Q*_Mg_, *Q*_Ca_ due to the elements H I, Mg II and Ca II, and cooling/heating *Q*_RT_ from the multigroup radiation transfer scheme.

### Simulation setup

Here, we differentiate between two simulation runs that have the same general structure, but different magnetic field configurations and a slightly different bottom boundary condition. We refer to them as Run I and Run II, according to the setup presented in this section. We used a 3D box with a size of 80 Mm (*x*) × 10 Mm (*y*) in the horizontal direction and a height of 32 Mm (*z*) for Run I, and a size of 80 Mm (*x*) × 10 Mm (*y*) in horizontal direction and a height of 34 Mm (*z*) for Run II. The resolution is 80 km × 80 km horizontally and 50 km vertically, corresponding to 1,000 × 125 × 640 grid cells for Run I and 1,000 × 125 × 680 grid cells for Run II. The difference in the vertical size comes from different convection zone depths: the convection zone is 2 Mm deep for Run I and 4 Mm deep for Run II. The atmosphere goes up to 30 Mm above the surface for both runs. The third Run that we present is a sheared configuration based on the setup of Run I, which is explained below. Apart from the bottom boundary condition that is used to drive the shearing motions, all properties that are presented here for Run I are also valid for the sheared configuration. The resolution used for the presented simulations is with a maximum of 50 km relatively low. In future setups, a higher resolution will be needed to study the small-scale dynamics of the simulated prominences in detail.

The 3D run was started from an evolved state of a two-dimensional (2D) run in both cases. The 2D run makes up the *x*–*z* plane and is later extended along the *y* direction for the 3D run. The initial conditions for the 2D runs were chosen such that they provide a dipped magnetic field, as shown in Extended Data Figs. 1 and 2. This initial magnetic field configuration was created by fixing a set of vertical magnetic field columns below the surface (up to 100 km below the surface) and performing a potential-field extrapolation into the atmosphere. For Run I, we chose six narrow columns for the vertical magnetic field. To get an overall higher field strength for Run II, we instead used four thicker columns while keeping the amplitude the same as in Run I. The columns in Run I all have the same spatial extend of 1.6 Mm in the *x* direction. The inner/outer vertical magnetic field columns for Run II each span 3.2 Mm/6.4 Mm. In both runs, the vertical magnetic field in the columns follows a Gaussian distribution in the horizontal (*x*) direction, with a maximum amplitude of *B*_*z*_ = 4.8 kG for the four (Run I)/two (Run II) outer columns and *B*_*z*_ = 2.88 kG for the two inner columns. From left to right, the columns have polarity +, +, −, +, −, − for Run I and +, −, +, − for Run II, such that the magnetic field resembles a quadrupolar structure in both cases. Their initial field strength is constant in the vertical direction within the convection zone. The horizontal field *B*_*x*_ is zero initially in the whole convection zone.

We aimed to create a stable dipped magnetic field configuration, such that mass can settle in the dips and stay there to form a prominence. To make the magnetic field shown in Extended Data Fig. 1 and 2 stable for extended lengths of time, we tied the field lines of the six/four vertical columns to the bottom of the convection zone. This is done by fixing the value of *B*_*z*_ in the first 10 (Run I)/20 (Run II) grid cells of the domain to the magnetic field distribution in the columns as described above. Outside of the vertical columns, *B*_*z*_ is set to 0 at the bottom boundary. Apart from *B*_*z*_, the only difference in the boundary conditions between Run I and Run II is the treatment of flows within the vertical magnetic field columns: for Run I, the bottom boundary is open for flows everywhere, inside and outside of the columns. For Run II, flows within the vertical magnetic field columns are suppressed by setting an asymmetric boundary condition for all velocity components. Outside of the columns, the boundary is open for flows. The bottom boundary condition for *B*_*x*_ (and *B*_*y*_ in the 3D run) is symmetric everywhere. The boundary conditions are periodic in both horizontal directions. The top boundary is open for outflows and a potential field extrapolation is used for the magnetic field.

After running the 2D calculation for a few hours of solar time, we picked one snapshot and stacked it in the *y* direction to create a 3D simulation. To break the symmetry in this direction, random fluctuations were introduced in the slices. The resulting magnetic field configuration is a 3D dipped magnetic arcade (Fig. 1 and Extended Data Fig. 5). No shearing motion was applied for Run I and Run II. Therefore, the initial magnetic field configuration includes no *B*_*y*_ component, in contrast to what is normally observed in prominences^1^. The resulting photospheric magnetic field and its evolution is shown Fig. 1 for Run I, in Extended Data Fig. 5 for Run II and in the Supplementary Videos 1 and 9. For the sheared setup of Run I, a shearing motion was applied to the *y* component of the velocity at the bottom boundary of the middle vertical magnetic field columns (Extended Data Fig. 1, second and fifth columns from the left side). Starting with a snapshot that already includes a formed prominence, the two columns were sheared with a velocity amplitude of *v*_*y*_ =−2 km s^−1^ and *v*_*y*_ =+2 km s^−1^. The shearing profile follows the same Gaussian distribution along the *x* axis as the magnetic field. This led to a shearing of the magnetic loops between the two middle columns. This shear is decreasing with height, such that the lower part of the prominence is sheared the most. With increasing height, the shear becomes weaker, and the sheared arcade smoothly transitions to the overlying coronal loops that are only weakly sheared. When the setup is sufficiently sheared, we start to continuously decrease the shear amplitude until a value of 100 m s^−1^ is reached, which is then kept constant. For the sheared setup, our goal is to compare the prominence properties with the non-sheared setup. We do not simulate the formation from scratch for the sheared prominence. Extended Data Fig. 3 shows the shear angle of the sheared setup along the polarity inversion line in dependence of height above the surface. The shear angle is calculated via $\alpha =\arctan ({B}_{x}/{B}_{y})$. The *B*_*x*_ component changes orientation at the null point, so that the shear angle changes sign at ~7 Mm.

Extended Data Fig. 4 shows the horizontally averaged magnetic field and the magnetic field in the prominence core as a function of height for all runs. The average magnetic field strength at the surface is ~350 G for Run I, ~500 G for Run II and ~400 G for the sheared setup. The magnetic field strength in the magnetic dips at roughly half the height of the prominence is ~35 G for Run I, ~60 G for Run II and ~45 G for the sheared setup. The average field at these heights is larger than the field in the prominence itself, being ~80 G in Run I and in the sheared setup and ~250 G in Run II. For the sheared case, we see that *B*_*y*_ > *B*_*x*_ over most of the prominence height along the polarity inversion line, but not when averaged over the full box width. This shows that the shear is stronger around the polarity inversion line than in the rest of the box.

In addition to the different used magnetic field configurations, we performed simulations in LTE and NLTE, as described in the previous subsection. Due to the long time it needs for the prominence seed to be ejected into the corona (7–10 h from the start of the 3D simulation), the formation process is only simulated in our LTE runs. NLTE runs for Run I and Run II are started from an evolved state of the already existing prominence, because of the higher computational needs of such runs. The LTE treatment uses the Chianti loss function^62^, the NLTE treatment the Carlsson and Leenaarts loss function^63^. The Shear setup is only simulated in LTE.

## Supplementary information

Supplementary Information Supplementary Figs. 1–14 and the captions for Videos 1–14.

Supplementary Video 1 Magnetogram for Run I, corresponding to Fig. 1a. Time evolution of the magnetogram at the *τ*_500_ = 1 surface for Run I, starting approximately 20 min before prominence formation.

Supplementary Video 2 Prominence formation in Run I, part I, corresponding to Fig. 2a. A 3D rendering of the prominence density, showing the beginning of prominence formation in Run I. The cool plasma that is injected along the small loop from the left side starts the formation process. The colouring of the plasma shows the logarithmic gas density (in grams per cubic centimetre). For plasma with a density below 10^−14^ g cm^−3^, the opacity is set to zero, such that the surrounding corona is not visible.

Supplementary Video 3 Prominence formation in Run II, part I, corresponding to Fig. 2b. A 3D rendering of the prominence density, showing the beginning of prominence formation in Run II. The cool plasma that is injected from below (at the polarity inversion line) starts the formation process. Draining events can also be seen towards the end of the movie. The colouring of the plasma shows the logarithmic gas density (in grams per cubic centimetre). For plasma with a density below 10^−14^ g cm^−3^, the opacity is set to zero, such that the surrounding corona is not visible.

Supplementary Video 4 Prominence formation in Run I, part II, corresponding to Fig. 2c–f. It shows how the prominence in Run I is fed via condensation of hot plasma that is flowing along the magnetic field lines onto the cool prominence structure, driven by a pressure drop at the cool prominence material. For better visibility, the density (top left) and horizontal velocity (top right) are averaged over the current line-of-sight, whereas the temperature (bottom left) and pressure (bottom right) are taken along one vertical slice of the box. Top right: the arrows show the line-of-sight averaged velocity field. Line-of-sight averaged magnetic field lines are added to the bottom left.

Supplementary Video 5 Prominence formation in Run II, part II, corresponding to Fig. 2c–f. It shows how the prominence in Run II is fed via condensation of hot plasma that is flowing along the magnetic field lines onto the cool prominence structure, driven by a pressure drop at the cool prominence material. For better visibility, the density (top left) and horizontal velocity (top right) are averaged over the current line-of-sight, whereas the temperature (bottom left) and pressure (bottom right) are taken along one vertical slice of the box. Top right: the arrows show the line-of-sight averaged velocity field. Line-of-sight averaged magnetic field lines are added to the bottom left.

Supplementary Video 6 Prominence dynamics in Run I, corresponding to Fig. 3. Prominence dynamics for Run I, seen from three directions. The movie starts at prominence formation time. Left: integrated density through the prominence from the side (integration along the *x* axis). Top right: integrated density through the prominence from the front (integration along the *y* axis). Bottom right: density along a horizontal cut through the simulation box, taken at a height of 12 Mm above the surface.

Supplementary Video 7 Prominence dynamics in Run II, corresponding to Fig. 3. Prominence dynamics for Run II, seen from two directions. The movie starts shortly before prominence formation. Left: integrated density through the prominence from the side (integration along the *x* axis). Right: integrated density through the prominence from the front (integration along the *y* axis).

Supplementary Video 8 Prominence dynamics in the Shear run, corresponding to Fig. 3. Prominence dynamics for the sheared setup of Run I, seen from three directions. Left: integrated density through the prominence from the side (integration along the *x* axis). Top right: integrated density through the prominence from the front (integration along the *y* axis). Bottom right: density along a horizontal cut through the simulation box, taken at a height of 12 Mm above the surface.

Supplementary Video 9 Magnetogram for Run II, corresponding to Extended Data Fig. 5a. Time evolution of the magnetogram at the *τ*_500_ = 1 surface for Run II, starting a few minutes before prominence formation.

Supplementary Video 10 Evolution of an injection event in Run II, corresponding to Supplementary Fig. 6. An example for two subsequent injection events in Run II, happening from below the null point. Left: integrated density from the side (line-of-sight is the *x* axis). Middle: integrated density from the front (line-of-sight is the *y* axis). Right: magnetogram at the surface (here *z* = 0 Mm). The vertical lines in the left/middle panel indicate over which range along the other horizontal axis the density in the middle/left panel is integrated.

Supplementary Video 11 Forces in the *z* direction and magnetic field components for an injection event in Run II, corresponding to Supplementary Fig. 7. Forces in the *z* direction and magnetic field components during the injection event that is shown in Supplementary Fig. 6. Top row: density (left), the *z* component of the momentum (middle) and the absolute value of the current density $\nabla \times \vec{B}$ (right). Middle row: *z* components of the Lorentz force (left), the pressure gradient force (middle) and the advective term (right) (see also equation (2) in the Methods). Bottom: *z* (left), *y* (middle) and *x* component (right) of the magnetic field. All quantities are averaged over the *y* axis in the region *y* = 5.8 − 7.4 Mm around the injection, as shown by the white vertical lines in the left panel of Supplementary Fig. 6. The black contours are taken at 5 × 10^−13^ g cm^−3^ of the integrated density that is shown in the top left panel. The solar surface is here at *z* = 4 Mm.

Supplementary Video 12 A 3D rendering of the magnetic field for an injection event in Run II, corresponding Supplementary Fig. 8, top. Surface magnetogram and 3D rendering of the magnetic field lines around the location where the dense blobs shown in Supplementary Fig. 6 get injected from the chromosphere into the corona. Rearrangements of the field lines around the null point are regularly visible. The black and white shading at the solar surface indicates the *B*_*z*_ there. The colouring of the field lines corresponds to *B*_*z*_ in units of gauss. The left black polarity at the surface is the same one as the red polarity in Supplementary Figs. 6 and 9.

Supplementary Video 13 A 3D rendering of the magnetic field and the chromospheric density for an injection event in Run II, corresponding to Supplementary Fig. 8, bottom. Surface magnetogram, 3D rendering of the magnetic field lines and volume rendering of the chromospheric plasma density around the location where the dense blobs shown in Supplementary Fig. 6 get injected from the chromosphere into the corona. During the time when chromospheric plasma is surging upwards, rearrangements of the magnetic field lines above the surface are visible. The colouring of the field lines corresponds to *B*_*z*_ in units of gauss. The opacity of the plasma density is adjusted such that only the upper chromosphere is visible.

Supplementary Video 14 Photospheric flux cancellation for an injection event in Run II, corresponding to Supplementary Fig. 9. Strong signatures of flux cancellation are visible at the photosphere during the injection events shown in Supplementary Fig. 6. Left: unsigned flux at the solar surface (*z* = 4 Mm in Supplementary Fig. 7) in the region around the negative polarity footpoint at *x* ≈ 40 Mm, *y* ≈ 7 Mm, measured within the black rectangle in the right panel. Right: magnetogram at the surface (*z* = 4 Mm in Supplementary Fig. 7).

## Data Availability

The data for snapshots of both Run I and II are available for download via the Edmond Open Research Data Repository of the Max Planck Society at 10.17617/3.8YEPQW (ref. ^64^).

## Code availability

The coronal extension of the MURaM Code^33^ is available for download via GitHub at https://github.com/NCAR/MURaM_main.
