# Supplementary material for: Self-consistent numerical simulations for the formation and dynamics of solar prominences
Source: Nat Astron. 2026 Apr 22;10(7):952–63. doi: 10.1038/s41550-026-02840-7 (PMC13379319; doi:10.1038/s41550-026-02840-7)
Supplement: Supplementary file 1 — Supplementary Figs. 1–14 and the captions for Videos 1–14. [file 41550_2026_2840_MOESM1_ESM.pdf]

nature astronomy

Supplementary information https://doi.org/10.1038/s41550-026-02840-7

Self-consistent numerical simulations for the formation and dynamics of solar prominences

In the format provided by the authors and unedited

Supplementary Text

This file includes Supplementary Text, including Supplementary Figures 1 to 14 and the captions for the Supplementary Videos 1 to 14.

Contents

S1 Energetics, radiative cooling and pressure **1**

S2 Thermal instabilities **4**

S3 Example of a cool injection **6**

S4 Prominence formation and mass supply **11**

S5 Prominence formation in current numerical simulations **20**

S6 Captions for Supplementary Videos **23**

S1 Energetics, radiative cooling and pressure

This section gives additional information on the energetics within the prominence and its surrounding transition region, as well as the siphon flows that are discussed in the main text (see Figure 2C-F). We present the heating and cooling rates, and show examples for the pressure and cooling distributions for LTE and NLTE runs.

The basis of the thermodynamic balance within the prominence can be seen in Supplementary Figure 1 for the LTE and NLTE versions of Run I. The left panels show the horizontal profiles of the terms dominating the energy evolution equation (see Equation 3 in the Methods Section), and the right panels show the corresponding composition of the radiative cooling term. The spatial profiles in each y-slice of the simulation were first centered on their respective center of mass before averaging a snapshot over the y-axis. These profiles change throughout the simulation, depending on the state of the prominence. Supplementary Figure 1 shows an example for an approximately vertical state of the prominence. For all runs, the cooling of the structure is largely dominated by the radiative losses (blue curve in the left panels, labeled as 'Q_{rad}'), which are divided into different contributions as shown in the right panels of the Figure: for the LTE runs, this includes the cooling and heating contribution from the radiative transfer scheme (labeled as RT losses 'Q_{RT}', blue curve in the right panels) and the contributions from a tabulated optically thin loss function for the corona (therefore labeled as optically thin losses 'Q_{thin}', orange curve). Additionally, the Carlsson & Leenaarts treatment in the NLTE runs includes tabulated line losses for H I ('Q_H', green curve), Mg II ('Q_{Mg}', red curve), and Ca II ('Q_{Ca}',

purple curve) that were calculated with a tabulated NLTE model (see the Methods Section for more information about the radiative losses). The prominence structure is heated by heat conduction ('$\nabla \cdot F_{con}$', orange curve) at the edges and by advection in the core ('$\nabla \cdot F_{adv}$', light blue curve). The NLTE simulation is run for a short time frame compared to the LTE simulation. During the NLTE runtime, the prominence partly drains to one side and does thus not reach a properly vertical state along the whole y-axis. Therefore, the advective term for the NLTE simulation looks more variable than in the LTE case. In contrast to the LTE simulation, the NLTE case includes a chromospheric backheating term ('Q_{back}', darkred curve) that additionally heats the prominence core.

While the NLTE runs are generally similar to the LTE runs, the distribution of temperatures in the prominence differs between the LTE and the NLTE simulations. We will address this in a follow-up paper about different radiative treatments for the setup of Run I. The prominence temperatures stated in the main text correspond to the temperatures found in the LTE runs.

To illustrate the cooling and heating at the edges of the prominence in this context, Figure 2 shows the dependence of the cooling and heating rates on the temperature for all pixels in the transition region in one snapshot of Run I. To select the transition region around the prominence, we consider all pixels with $1.5 \cdot 10^{-14}\,\mathrm{g\,cm^{-3}} \leq \rho \leq 1.5 \cdot 10^{-13}\,\mathrm{g\,cm^{-3}}$ over a height of $5\,\mathrm{Mm}$ above the surface. The cooling and heating rates include the contributions from the optically thin losses, the radiative losses from the RT scheme, the advective term, the conductive term, the resistive heating and the viscous heating. The corresponding mean/median curves in Supplementary Figure 2 show that the heating is on average smaller than the cooling in the temperature range $10^4\,\mathrm{K} \leq T \leq 3 \cdot 10^5\,\mathrm{K}$. This leads to a pressure drop as shown below in Supplementary Figure 3 and drives the siphon flow. Also, the first injected cool blobs that start the prominence formation have a transition region like this associated with them (see Supplementary Figure 5 in the next section).

To take a closer look at the radiative cooling and the Siphon flow described in the main text, Supplementary Figure 3 shows the radiative cooling and the pressure within the prominence. The maps on the left side show the distribution of the radiative cooling and those in the middle column the pressure for one vertical slice at one timestep for each run. The right panels of Supplementary Figure 3 show the corresponding distribution of the radiative cooling and the pressure along a horizontal slice, averaged over the y-axis as in Supplementary Figure 1. The height of the horizontal slices is indicated in the respective pressure maps on the left. The times of the four snapshots are chosen such that they represent an approximately vertical state of the prominence. The profiles on the right side show that the strong cooling at the edges of the prominence coincides with a pressure drop. This indicates that the radiative cooling drives this pressure drop, which then leads to a siphon flow from the corona onto the prominence. As

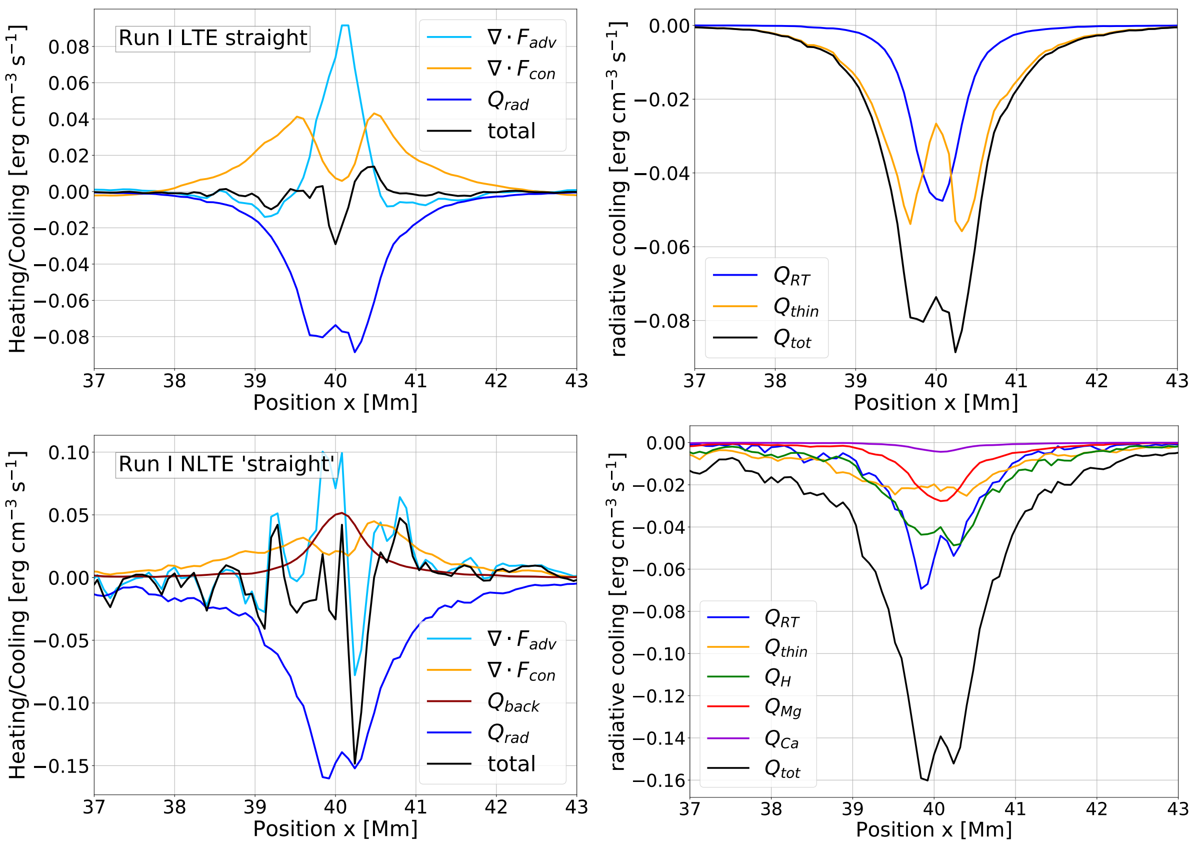

Supplementary Figure 1: Energetics of the prominence for Run I LTE and Run I NLTE. The top row shows the LTE version and the bottom row the NLTE version of Run I. The energy terms are shown along a horizontal slice at a height of 10 Mm above the surface, averaged over the y-axis and 60 minutes/5 minutes of simulation time for the LTE/NLTE run, respectively. Each vertical slice is centered on its respective center of mass before performing the y-average. The time frame is chosen such that the prominence is in an approximately straight vertical state. Due to the shorter runtime of the NLTE simulation, the simulated time frame does not include a prominence that is as vertical as for the LTE simulation. The advective term is therefore more variable. Left: The variously colored curves represent the dominating terms of the energy equation (Equation 3 in the Methods Section): the advective term ('$\nabla \cdot F_{adv}$', light blue), the conductive term ('$\nabla \cdot F_{con}$', orange), the total radiative cooling/heating ('Q_{rad}', blue) and the sum of all terms contributing to the energy equation (black). Right: Contributions to the radiative cooling/heating term (here the black curve): losses from the RT scheme ('Q_{RT}', blue), optically thin losses ('Q_{thin}', orange), Hydrogen line losses ('Q_H, green), Magnesium line losses ('Q_{Mg}, red) and Calcium line losses ('Q_{Ca}, purple). The latter three are only applicable for NLTE computations.

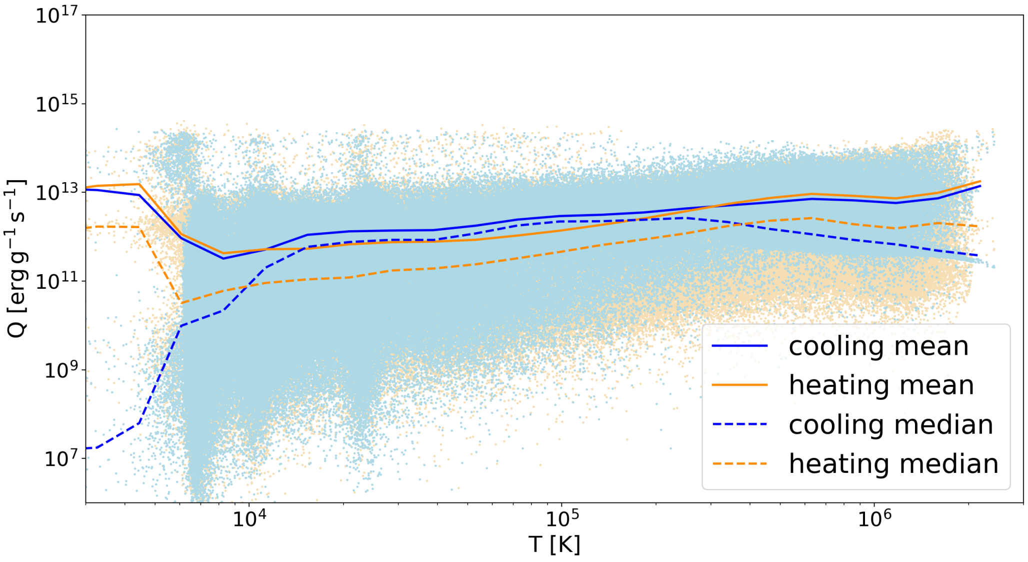

Supplementary Figure 2: Cooling and heating rates in the transition region around the prominence in Run I. The scatter plot shows the cooling (blue) and heating (orange) contributions for individual pixels in one snapshot of Run I. The transition region pixels are selected by taking all pixels with $1.5 \cdot 10^{-14}\,\mathrm{g\,cm^{-3}} \leq \rho \leq 1.5 \cdot 10^{-13}\,\mathrm{g\,cm^{-3}}$ above a height of $5\,\mathrm{Mm}$ above the surface. Included in the cooling and heating rates are the optically thin losses, the radiative losses from the RT scheme, the advective term, the conductive term, the resistive heating and the viscous heating (see also Supplementary Figure 1). The solid and dashed curves show the mean and median curves of the scattered points.

the mass has to follow the magnetic field lines, this pressure gradient creates an inflow from the chromosphere into the corona and then onto the prominence. As soon as the first dense plasma seed has settled in the magnetic dips, we can see the converging flows of hot plasma towards the prominence structure in the velocities, as shown in the Supplementary Videos 4 and 5, corresponding to Figure 2C-F in the main text.

S2 Thermal instabilities

Thermal instabilities have been found to play a role in the formation and dynamics of prominences in numerical simulations. Here we apply the corresponding criterion to snapshots of our simulations to test whether we find similar relations between the prominence material and unstable regions as in previous work.

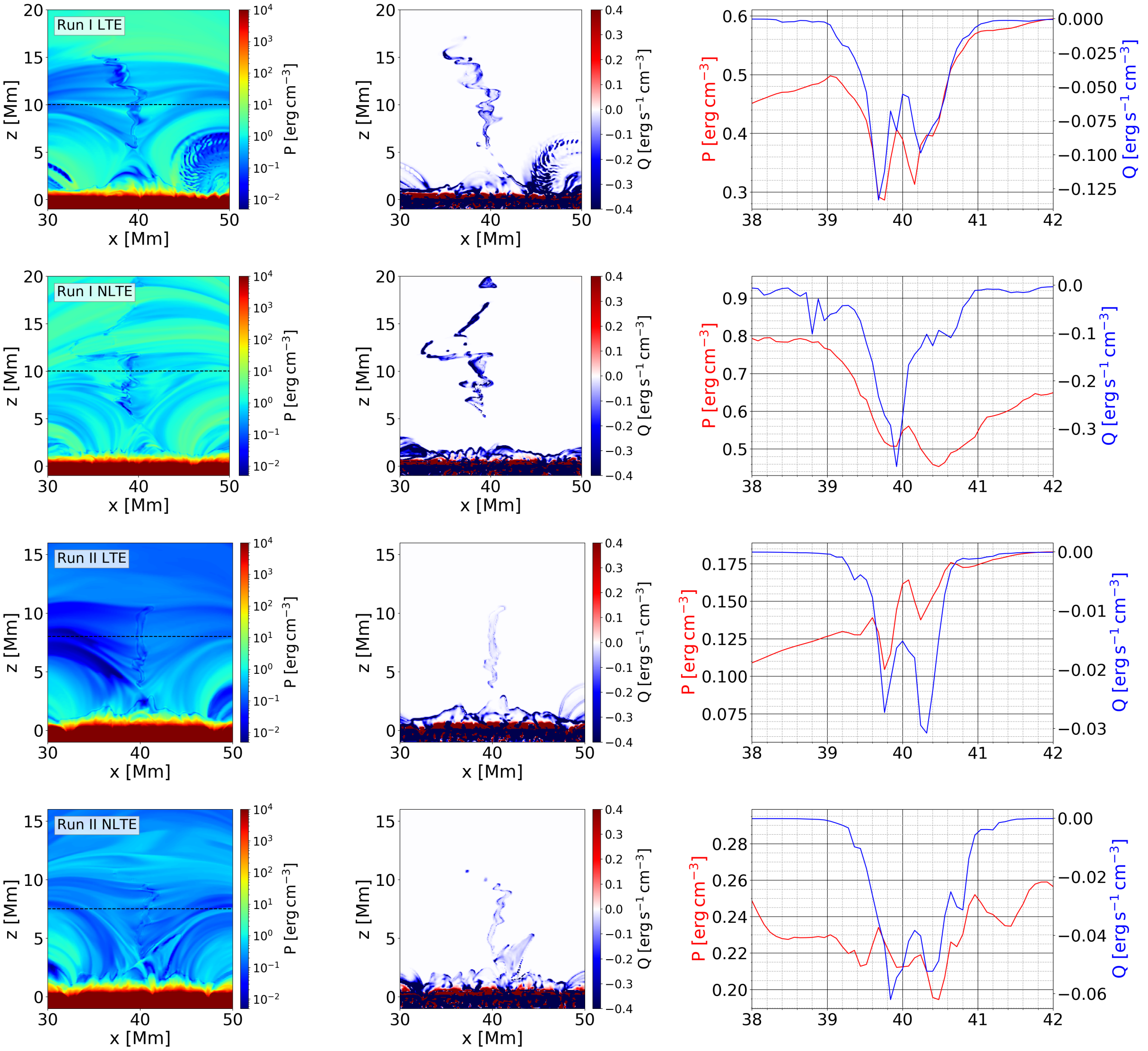

Supplementary Figure 3: Radiative cooling and pressure for one snapshot of Run I and Run II, as well as the corresponding NLTE runs. The color maps show pressure (left) and radiative cooling (middle) for a zoom-in on the prominence along a vertical slice of the shown snapshots. The plots on the right show the horizontal profiles of averaged pressure and radiative cooling along a horizontal slice. The height of the corresponding horizontal slices is indicated by the dashed black lines in the pressure maps on the left. Due to the shorter runtime of the NLTE simulations, the simulated time frames do not include a prominence that is as vertical as for the LTE simulations. Each row corresponds to one simulation, from top to bottom: Run I LTE, Run I NLTE, Run II LTE and Run II NLTE.

It has been shown that the isochoric instability criterion

$$C = k^2 - \frac{1}{\sigma}\left(\frac{\partial Q_H}{\partial T} - \frac{n_e n_H \partial \Lambda(T)}{\partial T}\right) < 0 \quad \text{(S.1)}$$

maps well onto the thermally unstable regions in the corona[1,2]. In equation S.1, the wavenumber $k = 2\pi/\lambda$ depends on the size λ of the condensation. σ is the Spitzer heat conductivity, Q_H denotes the heating terms in the simulation and the term $\frac{n_e n_H \partial \Lambda(T)}{\partial T}$ corresponds to the tabulated optically thin losses in the simulation (see also the description in the Methods Section). In the energy equation (equation 3 in the Methods Section), the heating terms and the radiative loss term from the radiative transfer scheme do not have a direct dependence on the temperature, therefore only the optically thin losses are considered to calculate C, similar to (Lu et al. 2024)[2]. The term $\frac{n_e n_H \partial \Lambda(T)}{\partial T}$ is calculated with the overlap interval that is used for the optically thin losses[3]. Supplementary Figure 4 shows C for a vertical slice through the prominence of Run I. We show here results for $\lambda = 4\,\mathrm{Mm}$, but qualitatively the unstable regions do not change much with varying λ. Supplementary Figure 4 shows that the transition region around the prominence is unstable according to $C < 0$. Similarly, Supplementary Figure 5 shows the criterion for a snapshot at the start of prominence formation in Run II. Similar to the already built-up prominence in Supplementary Figure 4, the first dense seed in Supplementary Figure 5 is surrounded by an unstable transition region. We do not see unstable regions along coronal loops before prominence formation as seen for e.g. the coronal rain simulated in (Lu et al. 2024)[2]. This suggests that thermal instabilities only start to set in after the formation was started by the first injection, because the transition region of the cool injections are thermally unstable.

S3 Example of a cool injection

This section presents more details on the dynamics of selected injection events. We present two subsequent injection events in Run II that happen at the beginning of the prominence formation process. Injection events like the ones presented below happen rather regularly before and after the prominence formation process starts, whereas the size and velocity of the blobs can vary. At the end of this Section, we show examples of two ejected blobs that do not contribute to the prominence mass.

For the selected event, Supplementary Figure 6 shows the integrated density from the side (left panel) and the front (middle panel) right after a dense blob has been ejected from the chromosphere into the corona. The right panel shows the corresponding magnetogram at the same time

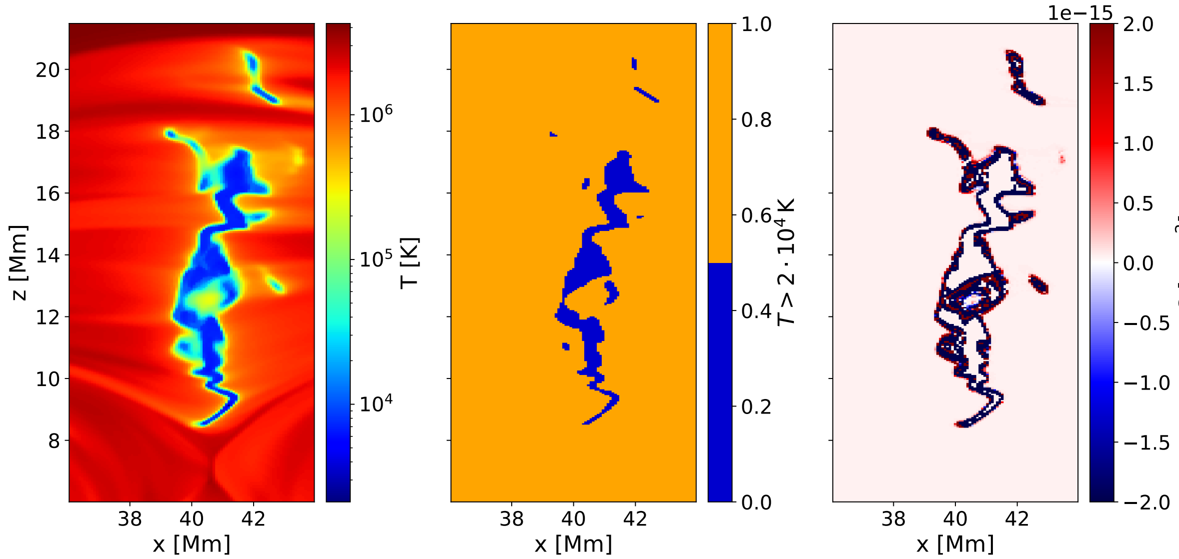

Supplementary Figure 4: Thermal instabilities for a snapshot of the prominence in Run I. A vertical slice from a snapshot of Run I when the prominence is fully formed, zoomed in to show the prominence material. Left: temperature. Middle: dual colorbar to differentiate between regions with $T > 2 \cdot 10^4$ K and $T < 2 \cdot 10^4$ K. The blue region roughly shows where the radiative losses from the RT scheme become dominant. The criterion calculated with the optically thin losses is valid for the orange region. Right: isochoric instability criterion C.

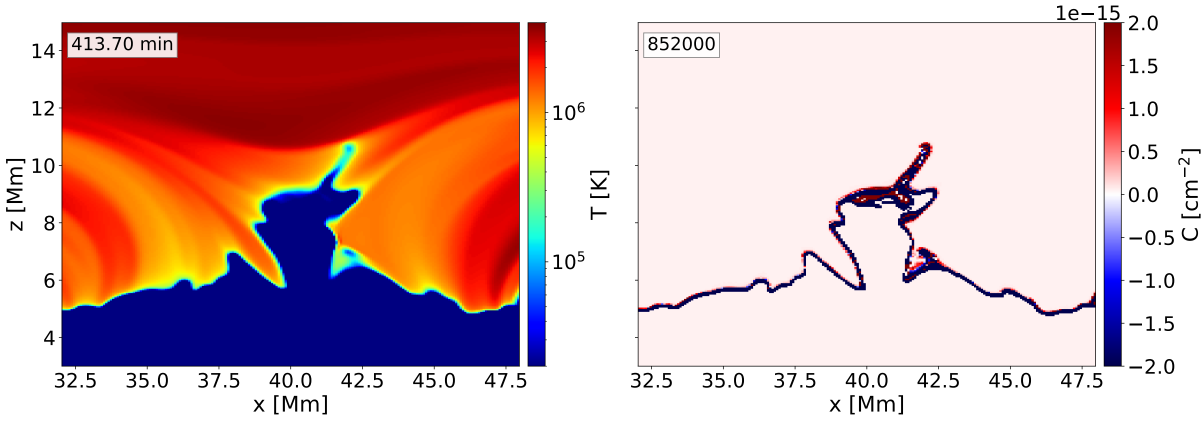

Supplementary Figure 5: Thermal instabilities for a snapshot during the prominence formation process in Run II. Similar to Supplementary Figure 4, but for a snapshot during prominence formation in Run II. At the shown time, a dense chromospheric seed is being ejected from the chromosphere into the corona. Left: temperature. Right: isochoric instability criterion C. The numbers in the top left corners show the time (left panel) and the iteration number (right panel) of the chosen snapshot.

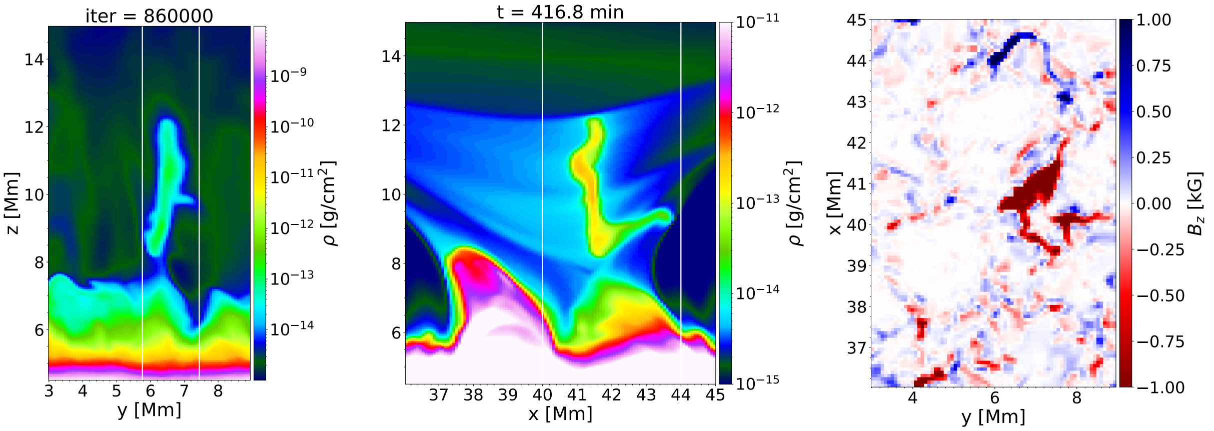

Supplementary Figure 6: An example for an injection event in Run II that is originating from below the Nullpoint. Shown is a snapshot of part of the domain taken when the dense chromospheric blob has already reached the dipped part of the fieldlines in the corona. Left: Integrated density from the side (the line-of-sight is the x-axis). Middle: Integrated density from the front (the line-of-sight is the y-axis). Right: magnetogram at the surface ($z = 0\,\mathrm{Mm}$). The white vertical lines in the left/middle panel indicate over which range along the other horizontal axis the density in the middle/left panel is integrated. Supplementary Video 10 shows an animation of this Figure.

for the region where the injection happens. Supplementary Video 10 follows the dense blob as it is ejected from the chromosphere until it reaches the corona. At the very beginning of the video, a previous ejection is seen at $x \sim 40$–$41\,\mathrm{Mm}$, $y \sim 3$–$4\,\mathrm{Mm}$, which reaches the corona but disappears shortly afterwards. After that, during $t \sim 409$–$415\,\mathrm{min}$, plasma surges up from the chromosphere at $x \sim 38$–$41\,\mathrm{Mm}$, $y \sim 4$–$7\,\mathrm{Mm}$. When viewed from the front (middle panel in Supplementary Figure 6 and Supplementary Video 10), a part of this plasma moves from below the dip towards the top left, while another part moves upwards through the Nullpoint into the corona. The mass of the plasma surge towards the top left is then also partly supplied to the dipped region in the corona while the mass is falling back down towards the Nullpoint. Once the plasma is in the corona, it rises slightly to around 6–8 Mm above the surface, marking the start of prominence formation. Right after this mass has reached the corona, a second surge happens close to the first one during $t \sim 414$–$423\,\mathrm{min}$ at $x \sim 37$–$40\,\mathrm{Mm}$, $y \sim 5$–$8\,\mathrm{Mm}$ that feeds more plasma into the dipped region. In the magnetogram in the right panel, signs of flux cancellation can be clearly seen during the time frame of the video, especially around the stronger red polarity at $x \sim 39$–$42\,\mathrm{Mm}$, $y \sim 6$–$8\,\mathrm{Mm}$ which lies below the ejected plasma blob that is shown in the left and middle panel of Supplementary Figure 6. This flux cancellation will be further discussed below in Figure 9.

Supplementary Figure 7 shows the forces in the z-direction (middle row) and the magnetic field

components (bottom row) for one snapshot of the first injection event. The top row shows the density, the z-component of the momentum and the absolute value of the current $\nabla \times \vec{B}$. The injection event is shown from the front, in the same way as the integrated density in the middle panel in Supplementary Figure 6. All quantities are averaged over the y-direction as indicated by the region between the two white lines in the left panel of Supplementary Figure 6. Supplementary Video 11 shows an animation of Supplementary Figure 7 for the time frame of the two injection events. For the snapshot shown in Supplementary Figure 7, the plasma below and left of the Null-point has started to rise. At this time, we can see an upward pressure gradient force in the lower part of the surging plasma and an upward directed Lorentz force in the top and left part of the surge. Later, at the beginning of the second injection ($t \sim 414\,\mathrm{min}$), strong upward-directed z-components can be seen in the video at the bottom of the surge at $x \sim$ 37–40 Mm in the Lorentz and the pressure gradient force. Both forces thus contribute to the dynamics of the injection. This is also the case for the two horizontal components of the forces, suggesting that the exact dynamics of the ejected chromospheric plasma is complicated. In the regions where the chromospheric plasma is surging upwards, changes in the magnetic field can be seen above the surface in all three components. In the y-component (middle bottom panel), the negative polarity region (blue) at $x \sim$ 38–41 Mm, $z \sim$ 4–8 Mm strongly changes shape during the first surge. In the z-component (bottom left), small inclusions of positive polarity (blue) can be seen in the negative polarity regions (red) at $x \sim$ 37–39 Mm, $z \sim$ 4–5 Mm below the location of the second surge, which move and then disappear toward the end of the video. Slight enhancements in the current density (bottom right) are visible at $z \sim$ 5–7 Mm in the lower part of the surges.

Supplementary Figure 8 shows a 3D rendering of the magnetic field lines around the location of the two injections. Here, the color of the field lines indicates B_z. The Null-point just below the magnetic dips is clearly visible. Supplementary Videos 12 and 13 show animations of the top and bottom panel of this Figure. At and above the surface, the red field lines (negative polarity) and the blue field lines (positive polarity) at the footpoints continuously interact with the surrounding field. The bottom panel of Supplementary Figure 8 additionally shows the density in the upper chromosphere to visualize when the injections happen. As in the video corresponding to Supplementary Figure 6 (Supplementary Video 10), the bottom panel of Supplementary Figure 8 (Supplementary Video 13) shows that plasma is shooting up from the chromosphere around the location of the left footpoint, which corresponds to the negative polarity at $x \sim$ 40–41 Mm in Supplementary Figure 6. The right footpoint corresponds to the positive polarity at $x \sim$ 43–45 Mm. Supplementary Figure 9 (Supplementary Video 14) shows that the unsigned photospheric flux in the region around the negative footpoint, as marked by the black rectangle in the right panel, is continuously decreasing during the time frame of the injections. Higher up in the chromosphere, changes in the magnetic field configuration around the footpoints are

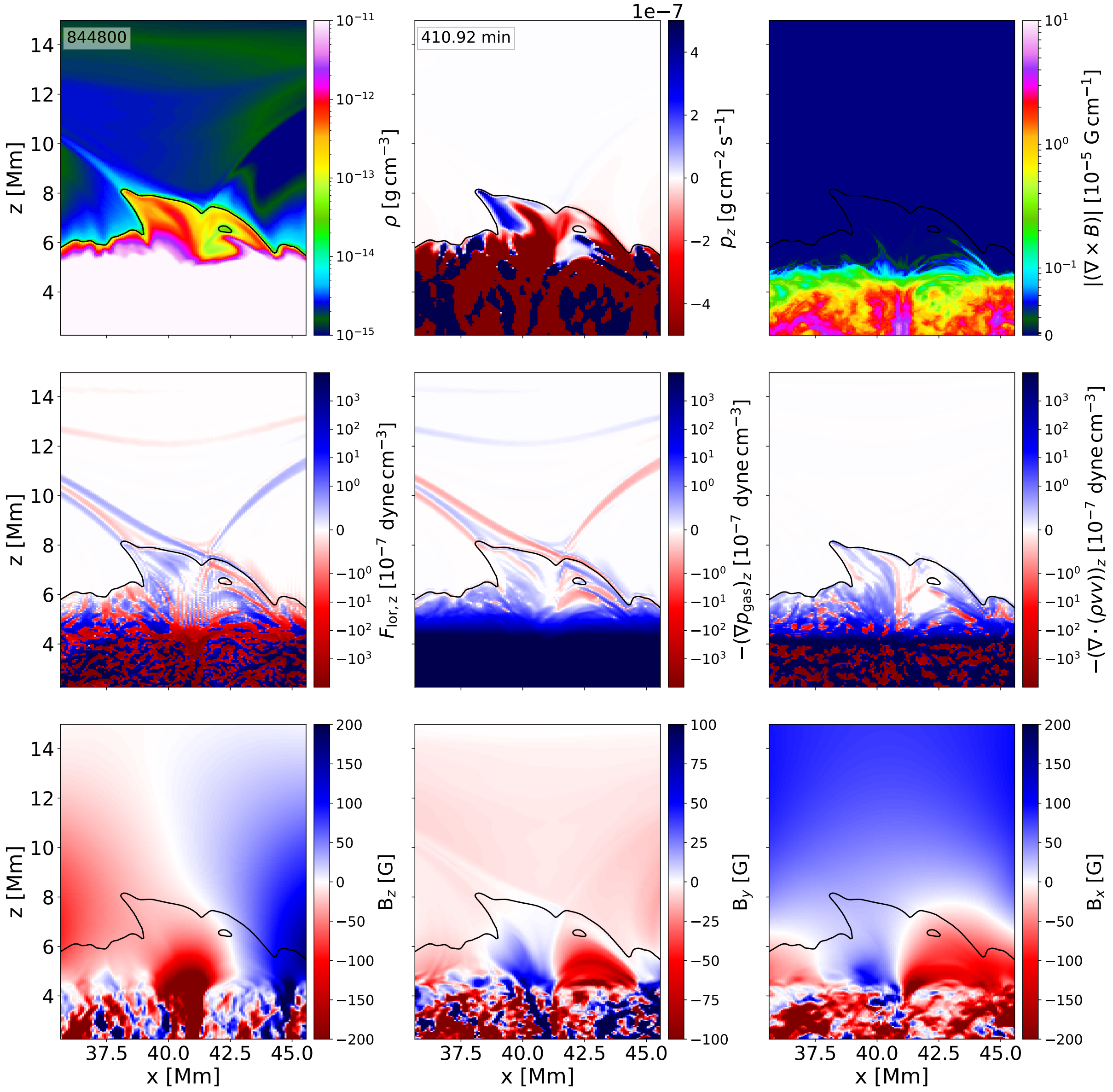

Supplementary Figure 7: Forces in the z-direction and magnetic field components for one snapshot during an injection event. Top row: density (left), the z-component of the momentum (middle) and the absolute value of the current density $\nabla \times \vec{B}$ (right). Middle row: z-components of the Lorentz force (left), the pressure gradient force (middle) and the advective term (right) (see also equation 2 in the Methods Section). Bottom: z- (left), y- (middle) and x-component (right) of the magnetic field. The shape of the shown injection event is also visible in Supplementary Figure 6. All quantities in this Figure are averaged over the y-axis in the region $y = 5.8–7.4\,\mathrm{Mm}$ around the injection, as indicated by the white vertical lines in the left panel of Supplementary Figure 6. The black contours are taken at $5 \cdot 10^{-13}\,\mathrm{g\,cm^{-3}}$ of the integrated density shown in the top left panel. The solar surface is at $z = 4\,\mathrm{Mm}$. Supplementary Video 11 shows an animation of this Figure.

visible in the 3D field lines. The blue inclusions in B_z from Supplementary Figure 7 during the second injection are well visible in the 3D field lines in the bottom panel of Supplementary Figure 8 (Supplementary Video 13): at $t \sim 414\,\mathrm{min}$, twisted blue field lines appear on the left side of the red footpoint, close to a smaller negative polarity at the photosphere. Until the end of the video, they seem to unravel, accompanied by the chromospheric plasma that surges upwards. After the chromospheric plasma is accelerated upwards from the surface, it reaches the corona through the Nullpoint. The video corresponding to the top panel of Supplementary Figure 8 (Supplementary Video 12) shows that rearrangements of the field lines around the Null-point happen regularly, allowing the chromospheric mass of the surges to reach the dipped region in the corona above the Nullpoint. Once in the corona, the cool prominence mass is supported against gravity by the Lorentz force, as shown in Supplementary Figure 10 for a longer-term average of the forces in the z-direction. This is also visible in the Supplementary Video 11 (corresponding to Supplementary Figure 7) once the chromospheric mass has moved through the Nullpoint into the corona. The Supplementary Videos 6 and 7 (corresponding to Figure 3 in the main text) show that these kind of surges happen regularly and at different locations along the x- and y-axis, but they can only reach and stay in the dipped region in the corona when they are magnetically connected to it.

In general, some of the ejected blobs feed mass to the prominence, while others do not contribute to the mass build-up. Supplementary Figure 11 shows three examples for ejected cool blobs before prominence formation starts in Run II. The first two shown blobs disappear after being injected, whereas the third one stays in the dipped region long enough to start the prominence formation process. This third blob corresponds to the first injection event shown above in Supplementary Figure 6. Supplementary Figure 12 shows histograms of the total velocity for the pixels belonging to each blob. The text boxes in Supplementary Figure 12 show the corresponding kinetic energy density, the total mass and the average total velocity of the three blobs. The disappearing blobs 1 and 2 have lower masses, a broader velocity distribution, and higher average velocities. This suggests that an injection should be slow and massive enough to stay in the magnetic dips. Generally, it can also happen for some injection events that a part of the prominence material is pushed out of the dipped region by injections with a larger momentum, inducing a draining event.

S4 Prominence formation and mass supply

In this section, we describe the prominence formation and the mechanisms that contribute to the mass growth of the prominence. Due to the longer runtime of the NLTE version of the code, the results in this section are shown for the LTE runs only. We start by qualitatively describing

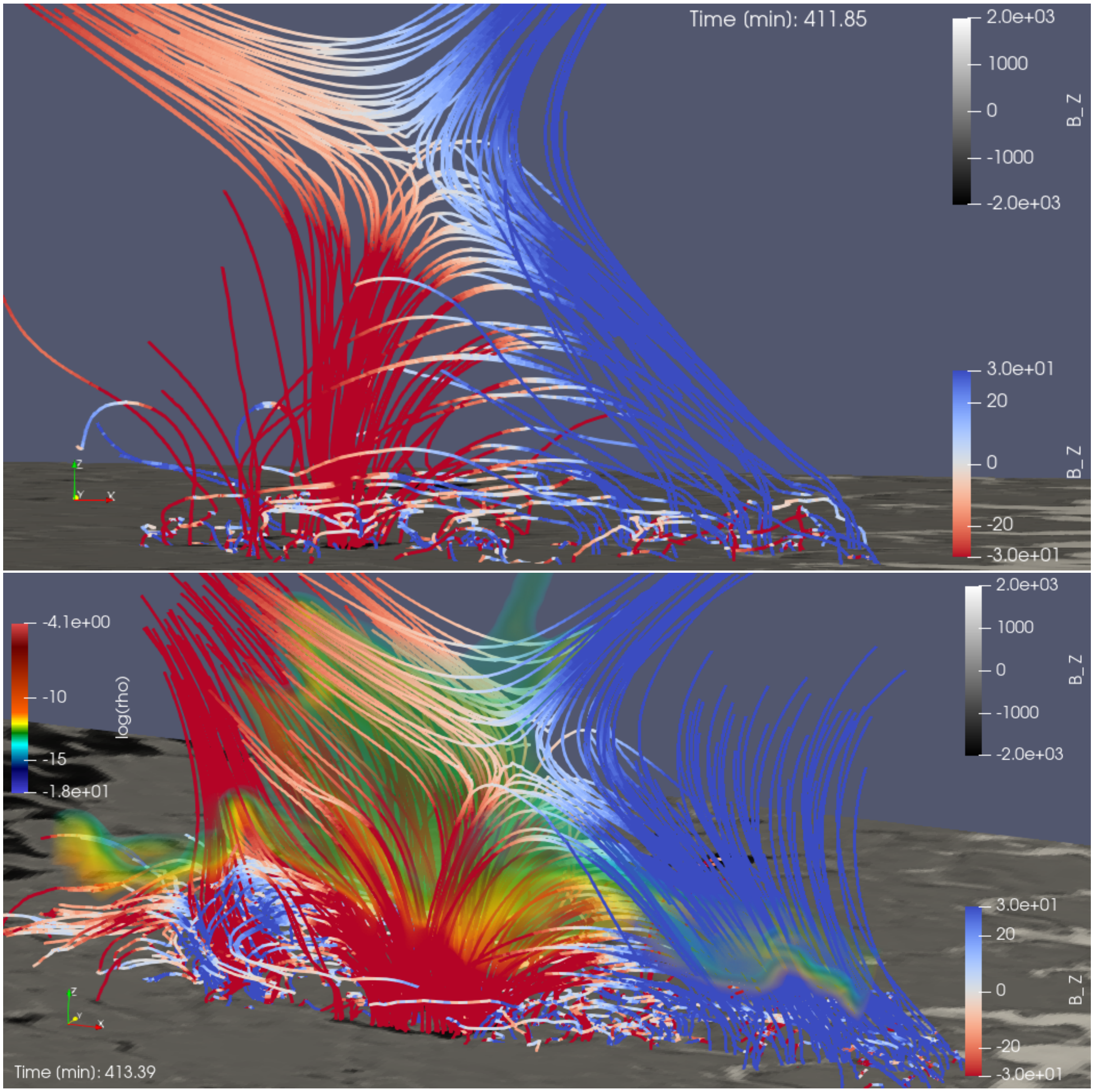

Supplementary Figure 8: Surface magnetogram and 3D rendering of the magnetic field lines around the location of an injection event. Shown is the region where the dense blobs from Supplementary Figure 6 get injected from the chromosphere into the corona. The bottom panel additionally includes a volume rendering of the plasma in the upper chromosphere. The top and bottom panels show the scene from two slightly different viewing angles. The black and white shading at the solar surface indicates the B_z there. The coloring of the field lines corresponds to B_z in Gauss. The left black polarity at the surface is the same one as the red polarity in Supplementary Figure 6 and Supplementary Figure 9. The bottom panel includes more seedpoints for the fieldlines compared to the top panel. Supplementary Videos 12 and 13 show an animation of the top and bottom panel of this Figure.

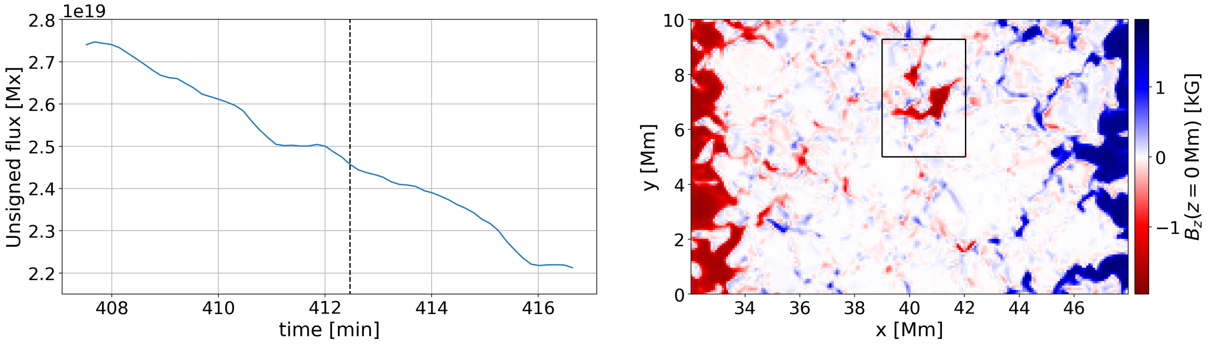

Supplementary Figure 9: Photospheric flux cancellation during an injection event. Shown is the region where the dense blobs from Supplementary Figure 6 get injected from the chromosphere into the corona. Left: Unsigned magnetic flux at the surface in the region around the negative polarity footpoint at $x \sim 40\,\mathrm{Mm}$, $y \sim 7\,\mathrm{Mm}$, measured within the black rectangle in the right panel. Right: Magnetogram at the surface ($z = 4\,\mathrm{Mm}$ in Supplementary Figure 7). Supplementary Video 14 shows an animation of this Figure.

the formation for both simulation runs in the first paragraph, which is then followed by more quantitative descriptions for each run individually.

Qualitative description As described in the main text, the process of prominence formation consists of different parts for both simulation runs. It is started by a random ejection of a dense plasma seed from the chromosphere into the magnetic dips in the corona (see Supplementary Section S3). This happens on a regular basis during the simulation, but it needs hours of simulated solar time until a large enough seed reaches and stays in the dip of the magnetic field configuration. For Run I, the cool ejections happen along the small loops on both sides of the prominence. The first seed originates from the left side at $t \sim 674\,\mathrm{min}$. For Run II, the cool ejections originate from the region below the magnetic dips: small blobs are ejected upward in discrete events from time to time. The ejection that starts prominence formation in Run II for the first time happens at $t \sim 412\,\mathrm{min}$.

In both runs, the cool seeds start the formation of the prominence. Almost as soon as the dense plasma seed settles in the magnetic dip, an inflow of plasma from the corona onto the prominence occurs along magnetic field lines (as described in Section S1). These inflows consist of hot gas with $T \approx 3 \cdot 10^5$–$10^6\,K$ that cools and condenses onto the edges of the prominence (see Figure 2F and Supplementary Videos 4 and 5). This process corresponds to the condensation model and it provides approximately 18–42 % of the mass of the prominence (see next paragraph). However, it is not the only mechanism that supplies the prominence with chromospheric plasma. Blobs of chromospheric plasma, similar to the one that initiated the prominence for-

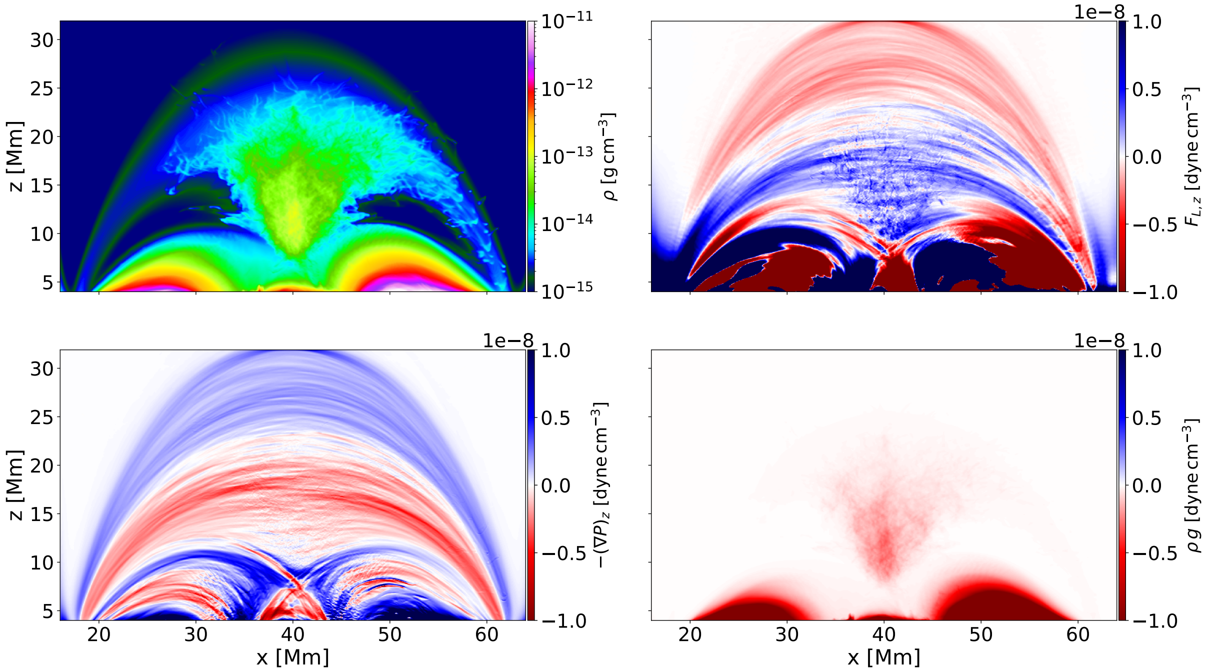

Supplementary Figure 10: Time-averaged density and force balance in the z-direction for Run I. Time-averages over 200 minutes for the density (top left) and the z-components of the Lorentz force (top right), the pressure gradient force (bottom left) and the gravitational force (bottom right). All panels are averaged over 0.8 Mm along the y-direction, corresponding to 10 vertical y-slices. In the time-averaged density, it is visible that the prominence is moving from side to side during the time frame considered here. Draining to both sides is visible, but draining to the right side dominates in this sample.

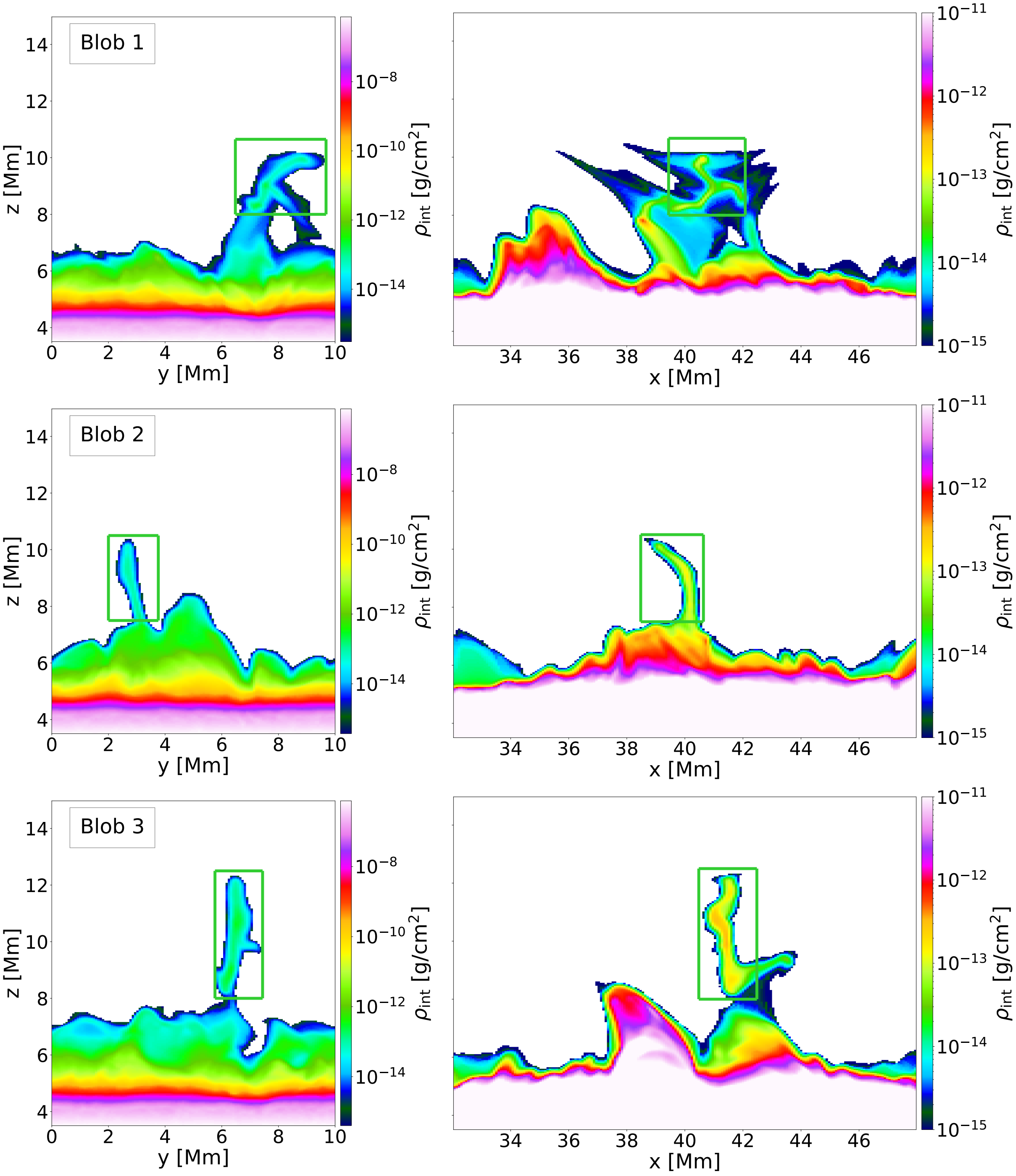

Supplementary Figure 11: Selection of three ejected chromospheric blobs in Run II. Blobs 1 (top), 2 (middle) and 3 (bottom) in integrated density from the side (left) and the front (right). The blobs are manually selected and marked by the green rectangles. Only plasma with $\rho > 10^{-14}\,\mathrm{g\,cm^{-3}}$ is shown and considered for the blob statistics in Supplementary Figure 12. Blob 3 is the same as shown in Supplementary Figure 6.

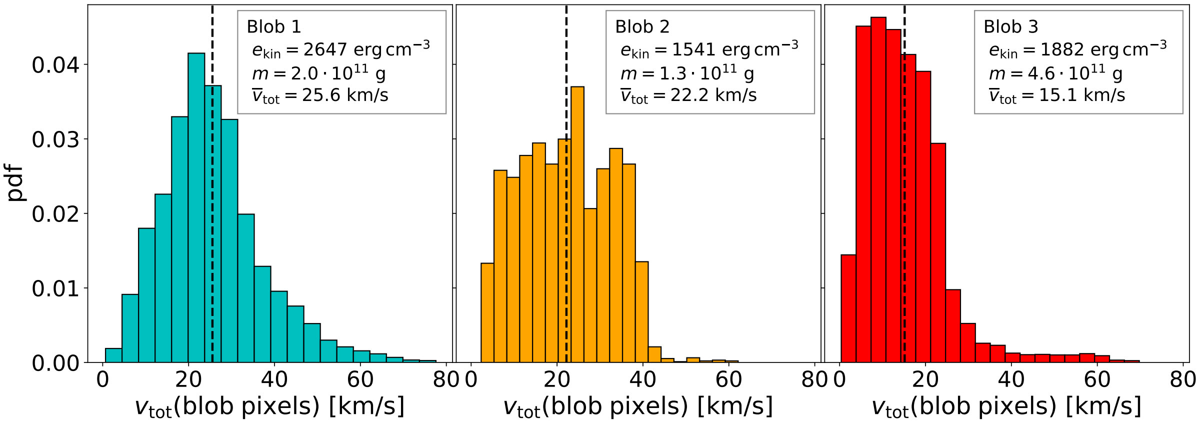

Supplementary Figure 12: Velocity distribution and masses for three ejected chromospheric blobs in Run II. Histograms of the total velocities of the pixels belonging to blobs 1,2 and 3 as shown in Supplementary Figure 11. The text boxes show the kinetic energy density, the total mass and the average total velocity of the corresponding blob. The dashed lines indicate the locations of the average velocity in the histogram.

mation, also get ejected during the further evolution of the prominence. Sometimes, the ejected plasma blobs reach and stay in the magnetic dips, thus adding mass to the existing prominence structure. This is also visible in the Supplementary Videos 6 and 7 which correspond to Figure 3 in the main text. When one of the blobs comes up and stays there, we can see an increase in the integrated density of the prominence (mostly in the left panel which shows the density integrated along the x-axis). This mechanism supplies approximately 58–82 % of the mass that circulates through the prominence (see next paragraph).

Quantitative estimate Supplementary Figure 13 and 14 quantify the approximate contribution of the two mechanisms, hot inflows, and cool injections, to the mass supply of the prominence, as well as the total mass of the prominence and the total amount of drained mass. Supplementary Figure 13 shows how the relevant mass flows are calculated. We calculate mass fluxes into and out of the prominence through the four surfaces of the shown white rectangular box: left, right, top, and bottom. As the prominence spans the whole box along the y-axis and the horizontal boundaries are periodic, there is no front/back component to the fluxes.

For flows through the white box surfaces, we distinguish three cases. First: all mass flows going in and out of the box, to get an estimate for the current total mass of the prominence. The measurement is started at prominence formation time, such that the background coronal density is not counted. Second: mass flows for which $T > 3 \cdot 10^5\,K$ holds, to estimate the mass supply by hot siphon inflows. Third: mass flows for which $T < 3 \cdot 10^5\,K$, to estimate the mass supply

by cool injections from the side/bottom. For the hot inflows, mass contributions from the left, right, and bottom surfaces are added (the contribution from the top surface is very small and can be neglected). In this way, flows that enter and leave the box without feeding the prominence are not counted for the mass budget. This mainly happens at the bottom edges of the box for Run I: plasma flows along the small loops left and right to the prominence. It enters the white box at the lower part of the side surfaces and leaves the box through the bottom surface. For the mass contribution by cool injections, we want to exclude the top part of the box to cut out the effect of draining events. Therefore, we introduce a vertical cut that divides the side surfaces for the cool flows in a bottom and a top part. This cut lies at 12 Mm above the surface for Run I and at 6 Mm above the surface for Run II. For the cool injections contribution, the bottom side parts and the bottom surface are then summed up.

For Run I, we see that the cool inflows along the side loops get thermalized: plasma that enters the box in the $T < 3 \cdot 10^5\,K$ regime leaves it at a higher temperature. Both the cool and hot flows are therefore corrected for this contribution. The blue rectangular box in Supplementary Figure 13 shows how the amount of drained mass is estimated: all plasma with $T < 9 \cdot 10^5\,K$ that is passing outwards through a blue surface is counted as drained mass (the bottom part of the box is not counted here, as depicted in the map, to exclude the dynamics happening at the bottom below the prominence). The draining temperature cut is set because we want to exclude the hot outflows along the open field lines on both sides of the magnetic arcade. The blue box is much wider than the white one because the draining measurement should not capture the oscillating motions of the prominence structure itself. All mass fluxes are corrected by subtracting background fluxes, which are estimated by doing the same measurement for a time frame of 100 minutes before the prominence starts to form. The size of the white and blue boxes is adjusted to the smaller prominence size for Run II. The measured background fluxes are averaged over time and then subtracted as a constant correction factor.

Supplementary Figure 14 shows the corresponding mass contribution after integrating the measured fluxes over time. The red and green lines show the contribution of hot and cool inflows. The total amount of draining that has happened until the respective time is shown with the solid blue line. The dashed black line represents the current total mass of the prominence (as estimated by the fluxes through the white box in Supplementary Figure 13). The limits for the total amount of mass that has circulated through the prominence at the respective time is calculated by taking the sum of total prominence mass and total drained mass (solid black line), and by taking the sum of the hot and cool inflows (dashed purple line).

The shown time frames start a few minutes before the prominences begin to form. The injection of the dense seed from the side/bottom that starts prominence formation happens at 674 minutes/412 minutes for Run I and Run II, respectively. The injection and draining events that

are described in the following can also be seen in the integrated density in the Supplementary Videos 6 and 7.

Total mass Looking at the dashed black line that shows the estimate for the prominence mass, we can see that, from the formation at 674 minutes, the prominence in Run I is growing in mass until it reaches a roughly constant value of $2–3 \cdot 10^{13}\,\mathrm{g}$ from around 820 minutes onwards. The full size of the prominence generally extends slightly over the edges of the white box, especially in the top part, where the prominence is widest. Therefore, left and right motions of the oscillating prominence are captured in the total mass measurement. At around 1130 minutes, the prominence mass slightly starts to grow again. The gain in mass during the simulation is also visible in the integrated density from the side (Supplementary Video 6, left panel). Shortly before the end of the simulated time frame, at around 1260-1340 minutes, the total prominence mass reaches a maximum of $8 \cdot 10^{13}\,\mathrm{g}$ before decreasing to $6 \cdot 10^{13}\,\mathrm{g}$. In Run II, the prominence is not stable over time. After the prominence starts to form at 412 minutes, its mass first increases to a maximum of around $6 \cdot 10^{12}\,\mathrm{g}$. At around 640 minutes, a big draining event starts that completely depletes the prominence mass (the dashed black line is not exactly zero afterwards because turbulent motions can still bring chromospheric material into the region enclosed by the white box, such that inflows are measured temporarily). At 890 minutes, the prominence starts to form anew, and stays at a roughly constant mass of $3.5 \cdot 10^{12}\,\mathrm{g}$ before disappearing again at 970 minutes. A new formation starts at 1090 minutes, shortly before the simulated time frame ends.

Draining The blue line in Supplementary Figure 14 shows the total amount of draining that has happened before the respective point in time. In both runs, the draining consists of discrete events that can happen to either side of the prominence. Because the height of the prominence in Run I is comparable to the height of the simulated box, Run I also shows draining events at the top blue surface in Supplementary Figure 13. For Run I, examples of draining events that can be seen in Supplementary Video 6 (corresponding to Figure 3 in the main text), happen on the left side at $t \approx 835–860\,\mathrm{min}$ (mid-sized draining) and at $t \approx 930–950\,\mathrm{min}$ (big draining), as well as on the right side at $t \approx 750–800\,\mathrm{min}$ (big draining) and $t \approx 970–1000\,\mathrm{min}$ (big draining). For Run II (Supplementary Video 7), example draining events can be seen on the left side at $t \approx 460–480\,\mathrm{min}$ (small draining), $t \approx 545–555\,\mathrm{min}$ (small draining) and $t \approx 700–720\,\mathrm{min}$ (big draining), as well as on the right side at $t \approx 490–500\,\mathrm{min}$ (small draining) and $t \approx 645–660\,\mathrm{min}$ (big draining).

Hot siphon inflows The contribution by hot inflows (red line) is continuous for both runs. Hot gas flows from the loop footpoints up to the magnetic dips, where they condense when converging onto the prominence structure. When the prominence is not draining, the siphon inflow is visible in the velocities (see Figure 2C-F in the main text and the Supplementary Videos 4 and 5). Also in the bottom panel of Supplementary Figure 14, it is visible for Run II that the siphon flows set in as soon as the prominence starts to build up, but vanish after the prominence disappears (there is hot plasma below the prominence that is flowing all the time along the small magnetic loops on both sides, but the flows that lead higher up into the corona only start after the first dense seed of the prominence appears.). An estimate for how much mass the hot inflows contribute to the prominence follows below.

Cool injections The ejection of cool plasma from the chromosphere into the corona happens in discrete events. Not all ejections contribute mass the the prominence: some ejected plasma blobs fall back to the surface without feeding the prominence (see also Supplementary Figures 11 and 12). For Run II, cold plasma blobs are mostly ejected from below the magnetic Nullpoint, i.e. from directly below the prominence. For Run II (Supplementary Video 7), examples of mass contributions via injection can be seen at $t \approx 430\,\mathrm{min}$ (shortly after formation, in integrated density visible at $x \approx 40\,Mm$,$y \approx$ 6–8 Mm), at $t \approx$ 560–580 min (shortly after a draining event; in integrated density we see that one part of the ejected material shoots out to the right side and does not supply mass to the prominence). For Run I (Supplementary Video 6), these ejections happen along the small magnetic loops on both sides of the prominence. Little jet-like events happen at the outer footpoints and drive a plasma flow along the magnetic field lines, either back to the surface or into the prominence structure. This can for example be seen in the Supplementary Videos 2 and 6 (corresponding to Figure 2A and Figure 3 in the main text) at the beginning of the prominence formation process. A description of an injection event in Run II is presented in Supplementary Section S3.

Total circulated mass From the discussion above, we can infer how much mass circulated in total through the prominence structure relative to the mass of the prominence. To calculate the total mass that flowed in and out of the prominence structure, we have two estimates. First, we add the current prominence mass (dashed black line in Figure 14) and the total amount of draining (blue line) to get the solid black line. This total mass shown by the solid black line could be underestimated because part of the prominence plasma is located in the region between the white and blue surfaces in Supplementary Figure 14: parts of the prominence are too far out to be counted as prominence mass, but are not leaving the blue surface and are therefore not counted as draining, either. The solid black line thus represents a lower limit for

the total circulated mass. To get a second estimate, we calculate the total circulated mass by adding the mass supply contribution from the hot and cool inflows (red and green lines). This is shown in the dashed purple line and represents an upper limit for the mass estimate. For Run I, the total circulated mass is ~ 2.7–$2.95 \cdot 10^{14}\,\mathrm{g}$ at 1300 minutes (80 minutes before the end of the simulated time frame). The mass of the prominence at this time is $8 \cdot 10^{13}\,\mathrm{g}$, so the total amount of mass that flowed through the structure is $3.4 - 3.6$ times the prominence mass at this time step. For Run II, the total amount of circulated mass after the first complete drainage of the prominence (at $t \approx 733\,\mathrm{min}$) is $1.4 \cdot 10^{13}\,\mathrm{g}$. The maximum mass of the prominence until this point is $6 \cdot 10^{12}\,\mathrm{g}$, so the ratio between the total drained mass and the maximum prominence mass is 2.3. When we consider also the next two reappearances of the prominence, the total circulated mass at the end of the simulation time (at 1135 minutes) is 3–$3.7 \cdot 10^{13}\,\mathrm{g}$, which is $6 - 7.4$ times the current mass of $5 \cdot 10^{13}\,\mathrm{g}$ at this time. This strong mass circulation is also noted in observations[4] and emphasizes how dynamic the prominence is.

Contributions to mass supply With these values of the total circulated mass, we can now estimate how much of this mass is supplied by the hot inflows and cool injections. To calculate the contribution of both mechanisms, we calculate the ratios of the supplied hot and cool mass relative to the total circulated mass as given by the dashed purple curve. For Run I, we average these ratios over the last 400 minutes of the simulation time and take the maximum and minimum value in this time frame to estimate the variation of the ratios. The resulting contribution of hot inflows is 33–41 % with an average of 37 %. Consequently, the contribution of cool injections in Run I is 59–67 % with an average of 63 %. In contrast to Run I, the prominence in Run II is unstable. For the ratios, we therefore take all time frames during which the prominence is present, starting from 100 minutes after the first formation. The resulting ratios for the hot siphon contribution is 18–42 % with an average of 30 %. The contribution of cool injections in Run II is thus 58–82 % with an average of 70 %.For both runs, the cool injections are thus contributing more mass than the hot siphon inflows. The ratios are roughly similar for Run I and Run II, with the tendency that Run II is somewhat more dominated by the cool injections than Run I.

S5 Prominence formation in current numerical simulations

Many numerical prominence simulations use the condensation model as a base for prominence formation[5–11]. An often used mechanism is evaporation-condensation: Localized static or stochastic heating is manually applied at the loop footpoints, which leads to evaporation of chromospheric plasma at the loop footpoints and thus to an increase in density at the top of the

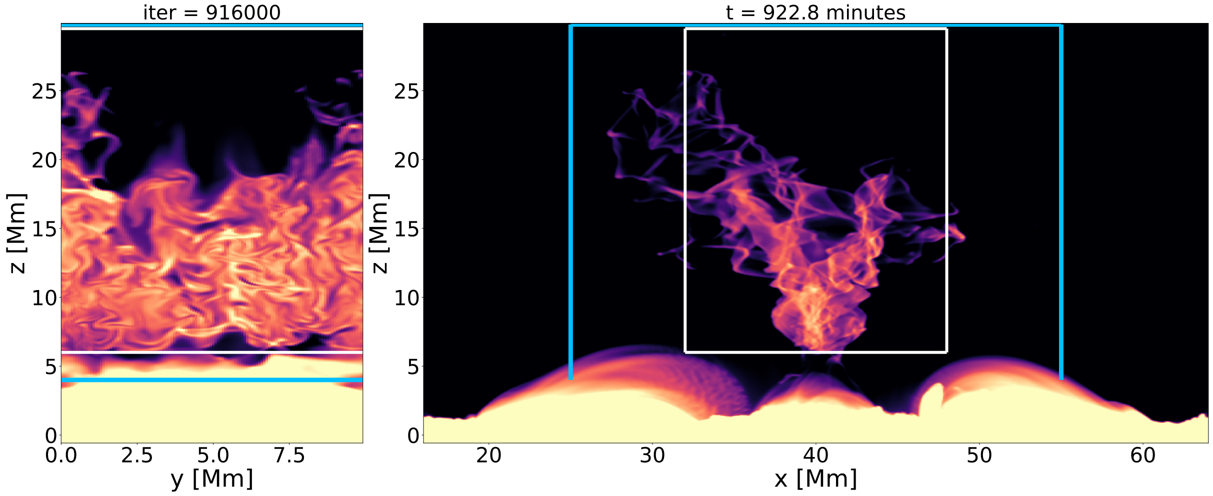

Supplementary Figure 13: Analysis of the mass flows into/out of the prominence (I) Integrated density of the prominence from the side (left panel) and the front (right panel) for one snapshot of Run I. The white rectangle defines the surfaces through which the mass flows for the total mass, the hot flows and cool flows are measured. The blue lines show the surfaces for measuring flows of draining events. The resulting time-integrated mass fluxes are shown in Supplementary Figure 14.

loop. Due to this increased density, plasma condensations start to form via thermal instabilities[12] or thermal non-equilibrium[13] at the top of the loop. The condensed plasma then either falls into pre-existing magnetic dips, or locally drags down the magnetic field lines to form new dips[14]. Donné and Keppens (2024)[11] recently showed that localized footpoint heating is not necessarily needed: in their setup, thermal instabilities are enough to drive siphon inflows that condense onto the prominence.

While we see condensation happening through siphon flows and thermal instabilities (see Supplementary Sections S1 and S3), the formation of our prominence is not started by a condensation mechanism. The formation starts when a cool and dense chromospheric plasma blob is ejected from the surface and gets stuck in the magnetic dips of our configuration. Only when this dense blob stays in the magnetic dips do we see the siphon flows setting in. The onset of the hot flows after the first injection is well visible in the Supplementary Videos 4 and 5 corresponding to Figure 2C-F in the main text. The formation of our prominence is thus started by injection, and the mass supply to the existing prominence structure is a combination of injection and condensation of the hot inflows.

The models by Kaneko and Yokoyama (2015)[15] and Jenkins and Keppens (2021)[16] form prominences by levitation and condensation: during the formation of the flux rope, denser plasma from the lower corona is lifted up with the magnetic field. Higher up it has a larger density than

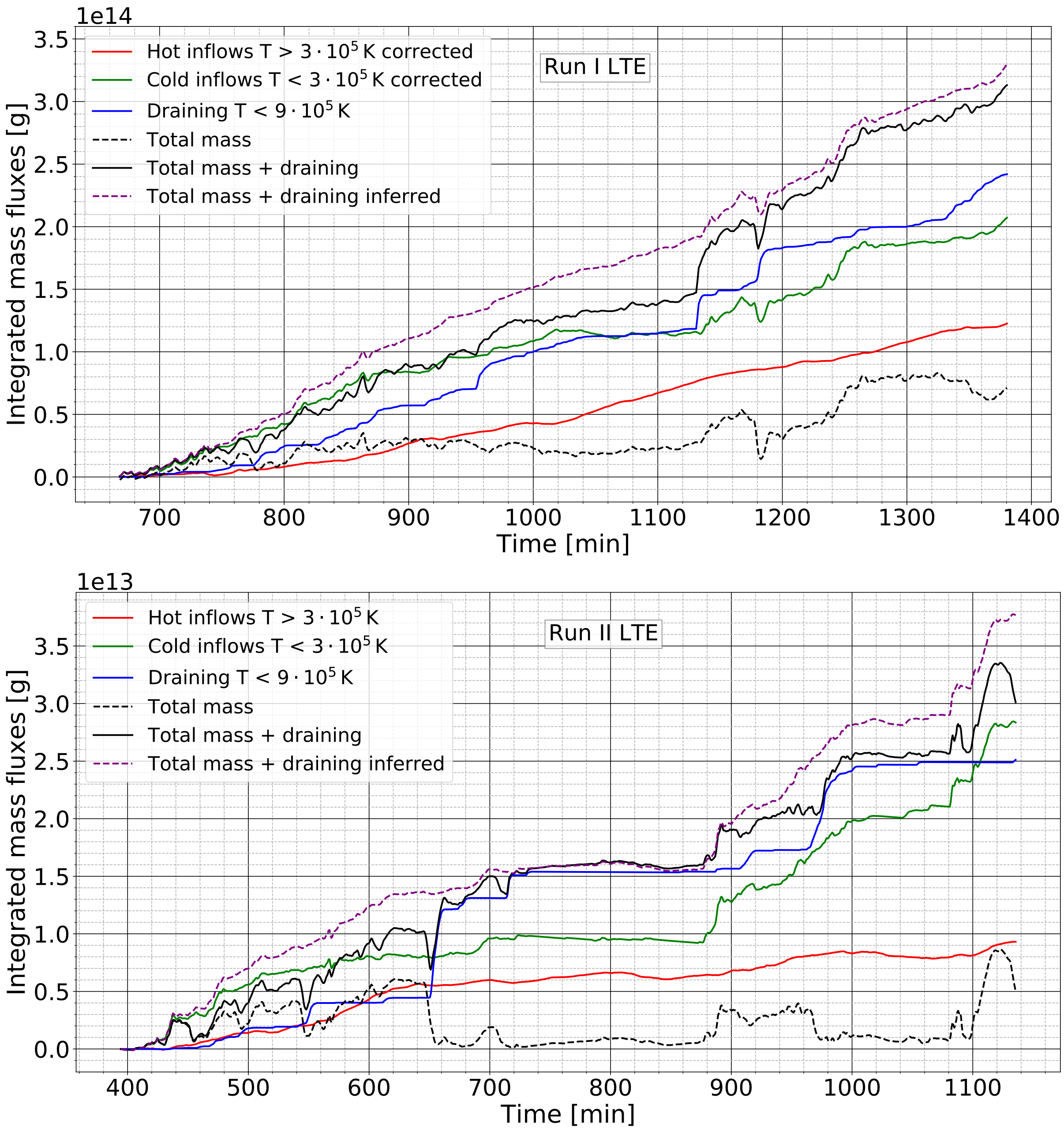

Supplementary Figure 14: Analysis of the mass flows into/out of the prominence (II) time evolution of the total prominence mass (black dashed line), the total drained mass (blue line), the total mass that circulated through the prominence (solid black and purple dashed line) and the mass supplied by hot (red line) and cool (green line) inflows onto the prominence, as illustrated by Supplementary Figure 13 and described in the text. The top panel shows Run I and the bottom panel shows Run II. The draining (blue line) measures mass going out of the prominence and has a negative contribution. We show the absolute value of the draining here to quantify the amount of mass that circulates through the prominence in total (solid black line).

the surroundings, leading to condensation via thermal instabilities. The second part is somewhat similar to our case, because denser mass from below is first brought higher up in the corona by another mechanism before condensation sets in.

There are also numerical simulations that form/feed prominences by the injection mechanism, with the difference that these simulations do not include a self-consistent treatment of the upper convection zone and photosphere. Huang (2021)[17] and Huang (2025)[18] formulated a model that explains both injections and condensation by the same physical mechanism. In Huang (2021)[17], the authors manually applied different heating profiles in the chromosphere and found that heating in the lower chromosphere leads to injections, while heating in the upper chromosphere leads to evaporation-condensation. In this manner, they found cases where injection and condensation can occur together to feed the prominence, depending on the applied heating profile. In Huang 2025[18], the applied heating was created self-consistently by including magnetic reconnection in the lower and upper chromosphere, confirming the unified model for injections and condensation found in[17].

Li (2025)[19] performed 2.5D simulations of flux emergence driven eruptions and found a model for flux-emergence-fed injections. The authors found that a filament channel is formed when the angle between the emerging and the pre-existing magnetic flux is 90°. The continued magnetic reconnection that forms the filament channel leads to the injection of cool plasma during the rise of the newly formed flux rope, feeding cool plasma into the channel. In comparison to our simulation, the injections in this work are driven by a global change of the magnetic field configuration, whereas the injections studied in Supplementary Section S3 are related to small-scale changes in the magnetic field driven by turbulent convective motions.

S6 Captions for Supplementary Videos

Supplementary Video 1: Magnetogram for Run I Video corresponding to Figure 1A in the main text: time evolution of the magnetogram at the $\tau_{500} = 1$ surface for Run I, starting approximately 20 minutes before prominence formation.

Supplementary Video 2: Prominence formation in Run I, part I Movie corresponding to Figure 2A in the main text: 3D rendering of the prominence density, showing the beginning of prominence formation in Run I. The cool plasma that is injected along the small loop from the left side starts the formation process. The coloring of the plasma shows the logarithmic gas density (in $\mathrm{g\,cm^{-3}}$). For plasma with a density below $10^{-14}\,\mathrm{g\,cm^{-3}}$, the opacity is set to zero, such that the surrounding corona is not visible.

Supplementary Video 3: Prominence formation in Run II, part I Video corresponding to Figure 2B in the main text: 3D rendering of the prominence density, showing the beginning of prominence formation in Run II. The cool plasma that is injected from below (at the polarity inversion line) starts the formation process. Draining events can also be seen towards the end of the movie. The coloring of the plasma shows the logarithmic gas density (in $\mathrm{g\,cm^{-3}}$). For plasma with a density below $10^{-14}\,\mathrm{g\,cm^{-3}}$, the opacity is set to zero, such that the surrounding corona is not visible.

Supplementary Video 4: Prominence formation in Run I, part II Video corresponding to Figure 2C-F in the main text: It shows how the prominence in Run I is fed via condensation of hot plasma that is flowing along the magnetic field lines onto the cool prominence structure, driven by a pressure drop at the cool prominence material. For better visibility, the density (top left) and horizontal velocity (top right) are averaged over the current line-of-sight, whereas the temperature (bottom left) and pressure (bottom right) are taken along one vertical slice of the box. The arrows in the top right panel show the line-of-sight-averaged velocity field. Line-of-sight averaged magnetic field lines are added to the bottom left panel.

Supplementary Video 5: Prominence formation in Run II, part II Video corresponding to Figure 2C-F in the main text: It shows how the prominence in Run II is fed via condensation of hot plasma that is flowing along the magnetic field lines onto the cool prominence structure, driven by a pressure drop at the cool prominence material. For better visibility, the density (top left) and horizontal velocity (top right) are averaged over the current line-of-sight, whereas the temperature (bottom left) and pressure (bottom right) are taken along one vertical slice of the box. The arrows in the top right panel show the line-of-sight-averaged velocity field. Line-of-sight averaged magnetic field lines are added to the bottom left panel.

Supplementary Video 6: Prominence dynamics in Run I Video corresponding to Figure 3 in the main text: Prominence dynamics for Run I, seen from three directions. The movie starts at prominence formation time. Left: Integrated density through the prominence from the side (integration along the x-axis). Top right: Integrated density through the prominence from the front (integration along the y-axis). Bottom right: Density along a horizontal cut through the simulation box, taken at a height of 12 Mm above the surface.

Supplementary Video 7: Prominence dynamics in Run II Video corresponding to Figure 3 in the main text: Prominence dynamics for Run II, seen from two directions. The movie starts shortly before prominence formation. Left: Integrated density through the prominence from the side (integration along the x-axis). Right: Integrated density through the prominence from the

front (integration along the y-axis).

Supplementary Video 8: Prominence dynamics in the Shear run Video corresponding to Figure 3 in the main text: Prominence dynamics for the sheared setup of Run I, seen from three directions. Left: Integrated density through the prominence from the side (integration along the x-axis). Top right: Integrated density through the prominence from the front (integration along the y-axis). Bottom right: Density along a horizontal cut through the simulation box, taken at a height of 12 Mm above the surface.

Supplementary Video 9: Magnetogram for Run II Video corresponding to Extended Data Figure 5A: time evolution of the magnetogram at the $\tau_{500} = 1$ surface for Run II, starting a few minutes before prominence formation.

Supplementary Video 10: Evolution of an injection event in Run II Video corresponding to Supplementary Figure 6 in the Supplementary Text: An example for two subsequent injection events in Run II, happening from below the Nullpoint. Left: Integrated density from the side (line-of-sight is the x-axis). Middle: Integrated density from the front (line-of-sight is the y-axis). Right: magnetogram at the surface (here $z = 0\,\mathrm{Mm}$). The vertical lines in the left/middle panel indicate over which range along the other horizontal axis the density in the middle/left panel is integrated.

Supplementary Video 11: Forces in the z-direction and magnetic field components for an injection event in Run II Video corresponding to Supplementary Figure 7 in the Supplementary Text: Forces in the z-direction and magnetic field components during the injection event shown in Supplementary Figure 6. Top row: density (left), the z-component of the momentum (middle) and the absolute value of the current density $\nabla \times \vec{B}$ (right). Middle row: z-components of the Lorentz force (left), the pressure gradient force (middle) and the advective term (right) (see also equation 2 in the Methods Section). Bottom: z- (left), y- (middle) and x-component (right) of the magnetic field. All quantities are averaged over the y-axis in the region $y = 5.8$–$7.4\,\mathrm{Mm}$ around the injection, as shown by the white vertical lines in the left panel of Supplementary Figure 6. The black contours are taken at $5 \cdot 10^{-13}\,\mathrm{g\,cm^{-3}}$ of the integrated density that is shown in the top left panel. The solar surface is here at $z = 4\,\mathrm{Mm}$.

Supplementary Video 12: 3D rendering of the magnetic field for an injection event in Run II Video corresponding to the top panel of Supplementary Figure 8 in the Supplementary Text: Surface magnetogram and 3D rendering of the magnetic field lines around the location where

the dense blobs shown in Supplementary Figure 6 get injected from the chromosphere into the corona. Rearrangements of the field lines around the Null-point are regularly visible. The black and white shading at the solar surface indicates the B_z there. The coloring of the field lines corresponds to B_z in Gauss. The left black polarity at the surface is the same one as the red polarity in Supplementary Figure 6 and Supplementary Figure 9.

Supplementary Video 13: 3D rendering of the magnetic field and the chromospheric density for an injection event in Run II Video corresponding to the bottom panel of Supplementary Figure 8 in the Supplementary Text: Surface magnetogram, 3D rendering of the magnetic field lines and volume rendering of the chromospheric plasma density around the location where the dense blobs shown in Supplementary Figure 6 get injected from the chromosphere into the corona. While chromospheric plasma is surging upwards, rearrangements of the magnetic field lines above the surface are visible. The coloring of the field lines corresponds to B_z in Gauss. The opacity of the plasma density is adjusted such that only the upper chromosphere is visible.

Supplementary Video 14: Photospheric flux cancellation for an injection event in Run II Video corresponding to Supplementary Figure 9 in the Supplementary Text: Strong signatures of flux cancellation are visible at the photosphere during the injection events shown in Supplementary Figure 6. Left: Unsigned flux at the solar surface ($z = 4\,\mathrm{Mm}$ in Supplementary Figure 7) in the region around the negative polarity footpoint at $x \sim 40\,\mathrm{Mm}$, $y \sim 7\,\mathrm{Mm}$, measured within the black rectangle in the right panel. Right: Magnetogram at the surface ($z = 4\,\mathrm{Mm}$ in Supplementary Figure 7).

References

1. Moschou, S., Keppens, R., Xia, C. & Fang, X. Simulating coronal condensation dynamics in 3d. *Adv. Space Res.* **56**, 2738–2759 (2015).

2. Lu, Z. *et al.* Periodic coronal rain driven by self-consistent heating process in a radiative magnetohydrodynamic simulation. *Astrophys. J. Lett.* **973**, L1 (2024).

3. Rempel, M. EXTENSION OF THE MURAM RADIATIVE MHD CODE FOR CORONAL SIMULATIONS. *Astrophys. J.* **834**, 10 (2017).

4. Liu, W., Berger, T. E. & Low, B. C. FIRST SDO/AIA OBSERVATION OF SOLAR PROMINENCE FORMATION FOLLOWING AN ERUPTION: MAGNETIC DIPS AND SUSTAINED CONDENSATION AND DRAINAGE. *Astrophys. J. Lett.* **745**, L21 (2012).

5. Antiochos, S. K., MacNeice, P. J., Spicer, D. S. & Klimchuk, J. A. The Dynamic Formation of Prominence Condensations. *Astrophys. J.* **512**, 985 (1999).

6. Antiochos, S. K., MacNeice, P. J. & Spicer, D. S. The Thermal Nonequilibrium of Prominences. *Astrophys. J.* **536**, 494 (2000).

7. Karpen, J. T., Antiochos, S. K. & Klimchuk, J. A. The Origin of High-Speed Motions and Threads in Prominences. *Astrophys. J.* **637**, 531 (2006).

8. Xia, C., Chen, P. F. & Keppens, R. SIMULATIONS OF PROMINENCE FORMATION IN THE MAGNETIZED SOLAR CORONA BY CHROMOSPHERIC HEATING. *Astrophys. J. Lett.* **748**, L26 (2012).

9. Fan, Y. MHD Simulation of Prominence Eruption. *Astrophys. J.* **862**, 54 (2018).

10. Jerčić, V., Jenkins, J. M. & Keppens, R. Prominence and coronal rain formation by steady versus stochastic heating and how we can relate it to observations. *Astron. & Astrophys.* **688**, A145 (2024).

11. Donné, D. & Keppens, R. Mass Cycle and Dynamics of a Virtual Quiescent Prominence. *Astrophys. J.* **971**, 90 (2024).

12. Parker, E. N. Instability of thermal fields. *Astrophysical Journal, vol. 117, p. 431* **117**, 431 (1953).

13. Klimchuk, J. A. The distinction between thermal nonequilibrium and thermal instability. *Sol. Phys.* **294**, 173 (2019).

14. Karpen, J., Antiochos, S., Hohensee, M., Klimchuk, J. & MacNeice, P. Are magnetic dips necessary for prominence formation? *Astrophys. J.* **553**, L85 (2001).

15. Kaneko, T. & Yokoyama, T. Numerical study on in situ prominence formation by radiative condensation in the solar corona. *Astrophys. J.* **806**, 115 (2015).

16. Jenkins, J. M. & Keppens, R. Prominence formation by levitation-condensation at extreme resolutions. *Astron. & Astrophys.* **646**, A134 (2021).

17. Huang, C. J., Guo, J. H., Ni, Y. W., Xu, A. A. & Chen, P. F. A Unified Model of Solar Prominence Formation. *Astrophys. J. Lett.* **913**, L8 (2021).

18. Huang, C. J., Ni, Y. W., Guo, J. H. & Chen, P. F. A Unified Model of Solar Prominence Formation with Self-consistent Heating. *Astrophys. J.* **991**, 215 (2025).

19. Li, X., Zhou, Y. & Keppens, R. Response of the solar atmosphere to flux emergence-with emergence-driven prominence formation. *Astron. & Astrophys.* **698**, A232 (2025).
